# Construction of a DNA damage repair gene signature for predicting prognosis and immune response in breast cancer

**DOI:** 10.3389/fonc.2022.1085632

**Published:** 2023-01-11

**Authors:** Yiming Chang, Zhiyuan Huang, Hong Quan, Hui Li, Shuo Yang, Yifei Song, Jian Wang, Jian Yuan, Chenming Wu

**Affiliations:** ^1^ Jinzhou Medical University, Shanghai East Hospital, Shanghai, China; ^2^ Research Center for Translational Medicine, Shanghai East Hospital, Tongji University School of Medicine, Shanghai, China; ^3^ Department of Breast Surgery, Shanghai East Hospital, Tongji University School of Medicine, Shanghai, China; ^4^ Department of Gynaecology and Obstetrics, Shanghai East Hospital, Tongji University School of Medicine, Shanghai, China; ^5^ Department of Medical Imaging, Shanghai East Hospital, Tongji University School of Medicine, Shanghai, China; ^6^ Department of Biochemistry and Molecular Biology, Tongji University School of Medicine, Shanghai, China; ^7^ Department of Pharmacy, Shanghai Pudong New Area People's Hospital, Shanghai, China; ^8^ Ji’an Hospital, Shanghai East Hospital, Ji’an, China

**Keywords:** breast cancer, prognostic model, immune filtration, immune response, DNA damage repair

## Abstract

DNA damage repair (DDR) genes are involved in developing breast cancer. Recently, a targeted therapeutic strategy through DNA repair machinery, including PARPi, has initially shown broad development and application prospects in breast cancer therapy. However, few studies that focused on the correlation between the expression level of DNA repair genes, prognosis, and immune response in breast cancer patients have been recently conducted. Herein, we focused on identifying differentially expressed DNA repair genes (DEGs) in breast cancer specimens and normal samples using the Wilcoxon rank-sum test. Biofunction enrichment analysis was performed with DEGs using the R software “cluster Profiler” package. DNA repair genes were involved in multivariate and univariate Cox regression analyses. After the optimization by AIC value, 11 DNA repair genes were sorted as prognostic DNA repair genes for breast cancer patients to calculate risk scores. Simultaneously, a nomogram was used to represent the prognostic model, which was validated using a calibration curve and C-index. Single-sample gene set enrichment analysis (ssGSEA), CIBERSORT algorithms, and ESTIMATE scores were applied to evaluate the immune filtration of tumor samples. Subsequently, anticarcinogen sensitivity analysis was performed using the R software “pRRophetic” package. Unsupervised clustering was used to excavate the correlation between the expression level of prognostic-significant DNA repair genes and clinical features. In summary, 56 DEGs were sorted, and their potential enriched biofunction pathways were revealed. In total, 11 DNA repair genes (*UBE2A*, *RBBP8*, *RAD50* , *FAAP20*, *RPA3*, *ENDOV*, *DDB2*, *UBE2V2*, *MRE11* , *RRM2B*, and *PARP3* ) were preserved as prognostic genes to estimate risk score, which was applied to establish the prognostic model and stratified breast cancer patients into two groups with high or low risk. The calibration curve and C-index indicated that they reliably predicted the survival of breast cancer patients. Immune filtration analysis, anticarcinogen sensitivity analysis, and unsupervised clustering were applied to reveal the character of DNA repair genes between low- and high-risk groups. We identified 11 prognosis-significant DNA repair genes to establish prediction models and immune responses in breast cancer patients.

## Introduction

Breast cancer is one of the most prevalent malignant diseases among women, leading to high medical costs yearly. More than two million new cases of breast cancer were diagnosed in 2020, according to the World Health Organization (WHO) (1). Based on its pathophysiology, breast cancer is a heterogeneous malignancy that is subdivided according to histological and molecular characteristics; the outcomes of treatment and prognosis are different for each of these subtypes (2–4). Perou et al. (5) reported that breast cancer cases could be classified into four intrinsic types based on gene expression profiles; each of them showed different characteristics of drug resistance, metastasis, and other characteristics. Subsequently, the number of intrinsic types was revised into six (6), which were basal-like, ERBB2+, normal breast-like, luminal subtype C, luminal subtype B, and luminal subtype A. Therefore, the potential prognostic predictors of breast cancer deserve exploration due to their heterogeneity.

DNA damage and DNA damage repair (DDR) play key roles in breast cancer progression. Some known types of DNA damage include single-strand breaks (SSBs), double-strand breaks (DSBs), base mismatches, pyrimidine dimers, and interstrand crosslinks. Different DNA repair mechanisms are applied to amend these DNA damage subtypes, including base excision repair (BER), nucleotide excision repair (NER), mismatch repair (MMR), homologous recombination repair (HRR), and non-homologous end joining (NHEJ) (7, 8). The balance between DNA damage and DDR systems maintains genome integration and stability. The defects or dysfunction of the DDR system leads to the occurrence and drug resistance of breast cancer. Mutations of the *BRCA1* and *BRCA2* genes in the germline were considered resources of genetic susceptibility for breast cancer (9). Approximately 50%–80% of hereditary breast cancer cases involve *BRCA1* or *BRCA2* mutations.

Furthermore, 30% of breast cancer patients without heredity were found to have methylation of the *BRCA1* and *BRCA2* promoter or dysfunctional upstream pathways, leading to descending levels of *BRCA1* and *BRCA2* (10, 11). DDR-targeted treatment has shown a significant improvement in progression-free survival in breast cancer patients. One of the most famous DDR-targeted drugs is the poly ADP-ribose polymerase inhibitor (PARPi), which can interact with a key upstream DNA repair enzyme, PARP.

DDR defect is cell damage, which may lead to genome distortion and malignant transformation. In contrast, the reduced DNA repair ability of cancer cells distinguished them from normal cells, which could be a character for target drug design (12). DDR genes provided doctors with broader treatment options for breast cancer patients (13). For instance, DDR polymorphism was independent of the treatment response of PD-1/PD-L1 inhibitors, while it was correlated with tumor mutation burden (14, 15). Harmful DDR mutations might abrogate the resistance of platinum-based treatment schemes for tumor tissues (16). Alteration of DDR genes might affect the prognosis of patients with breast cancer. However, few studies focused on the correlation between breast cancer prognosis and the expression level of DDR genes. The RNA-sequencing data were downloaded from The Cancer Genome Atlas (TCGA). The prognosis-related DDR genes were screened using Cox regression with the Wald *X* ^2^ test. The prognostic model was established based on a risk score calculated by sorted DDR genes and other clinical features. Generally, 11 prognostic-significant DDR genes were identified to establish a prediction model for patients with breast cancer. The sorted prognostic genes could be potential targets for novel breast cancer therapeutics.

## Methods

### Acquisition of data from the TCGA

The RNA-sequencing (RNA-seq) data of breast cancer specimens and the corresponding normal breast samples were downloaded from the TCGA database (https://portal.gdc.cancer.gov/), a famous cancer genomics database. The gene expression levels of 1,104 breast cancer specimens and 113 correspondent normal breast samples with their clinical information were involved in this research. Fragments per kilobase of exon model per million mapped fragments (FPKM) were used to normalize the RNA-seq data. Clinical data from breast cancer patients were downloaded from the TCGA database and integrated into the expression matrix utilizing the Perl software (**Supplementary Table 1**). Totally, 219 DNA repair genes (17) were selected to screen the gene expression profiles and establish a prognostic model.

### Identification of DEGs and enrichment analysis of biofunction

Differentially expressed genes (DEGs) were screened by the Wilcoxon rank-sum test with false discovery rate (FDR) correction. FDR <0.05 and |log2 fold change (logFC)| >0.5 were set as the cutoff points. The screened DEGs were presented through a volcano plot, heatmap, and box plot. Functional enrichment analyses, including Gene Ontology (GO) and Kyoto Encyclopedia of Genes and Genomes (KEGG), were performed using the R software “cluster Profiler” package (18). FDR <0.05 was established as the cutoff point to recognize significant items.

### Establishment of the Cox regression model

The survival time and status of breast cancer patients from the TCGA database were integrated into the expression matrix using Perl software. The level of expression of DNA repair genes from each breast cancer specimen with the survival of their patients was involved in univariate Cox regression. The Wald *X* ^2^ test for each variable was performed. Genes with *P <*0.05 were considered significant in prognosis. All prognosis-significant genes were applied to construct a multivariate Cox regression model, which was optimized by AIC value in a stepwise method to avoid overfitting. Each patient risk score was calculated according to the expression level, and the coefficient of each gene remained in the optimized multivariate Cox regression model. Risk score $=h_{0}\left(\text{t}\right)\text{exp}(\sum_{j=1}^{n}{\text{Coef}}_{j}\times {\text{X}}_{j})$ where *n* = quantity of sorted genes, Coef*_j_* is the coefficient of each DNA repair genes, *X_j_* is the relative expression level of each DNA repair gene, *h*_0_(*t*) is the baseline risk function. Subsequently, the risk score was included in the prognostic model with other clinical characteristics using another multivariate Cox regression analysis.

### Analysis of the risk score characteristics of breast cancer patients

Breast cancer patients were stratified into high- and low-risk groups using a median risk score. The survival of patients between these groups was compared using the Kaplan–Meier analysis with the log-rank test. Survival curves for the high- and low-risk groups were drawn utilizing the R software “survminer” package. A risk curve was drawn to show the distribution of risk scores for each breast cancer patient. Patients with risk scores less than the median were presented as green dots, while patients with risk scores higher than the median were shown as red dots. The correlation between risk score and the lifetime of each patient was revealed using the survival state plot. Based on data records, alive patients were displayed as green dots, and dead patients were shown as red dots. The risk heatmap revealed the expression level of prognostic DNA repair genes between the low- and high-risk groups.

### Receiver operating characteristic curve analysis

The feasibility of prognostic prediction of independent risk factors, including risk score and other clinical characteristics, was investigated using the receiver operating characteristic (ROC) curve with an area under the curve (AUC), which was drawn using the package “survivalROC” of the R software. The AUC of each prediction variable was compared at 1, 3, and 5 years. The AUC ranged from 0.5 to 1. The larger AUC indicates better prediction feasibility of the variable.

### Correlation analysis between clinical features and prognostic DNA repair genes

Correlations between the expression level of significant prognostic DNA repair genes and clinical characteristics such as gender, race, age, estrogen receptor, progesterone receptor, *HER2* receptor, clinical stage, and T, M, and N stages were evaluated using *t*-test or Kruskal–Wallis test which depends on the number of categories of the clinical feature. The risk score of each type was also compared. The expression level of prognostic DNA repair genes in each category of clinical features was presented utilizing a box plot.

### External validation of risk score

The breast cancer sample gene expression level data matrix with their clinical information data was downloaded from the Gene Expression Omnibus (GEO) database (GSE20685) to validate the prognostic model constructed using TCGA data. The risk score of each breast cancer patient was calculated according to the formula constructed before. Time-dependent ROC curves were used to measure the feasibility of prognosis prediction for risk score and other clinical features. Breast cancer patients in the GSE20685 dataset were classified into high- and low-risk groups based on the median risk scores, whose survival was compared using the Kaplan–Meier analysis and log-rank test. Univariate and multivariate Cox regression analyses were applied to reveal whether the risk score is an independent prognostic predictor.

### Establishing and validating the nomogram

Seven prognostic indicators, namely, gender, age, estrogen receptor status, progesterone receptor status, pathologic stage, T and N stages, and risk score calculated by DNA repair genes for prognostic prediction, were selected to establish the nomogram. Points for each prognostic factor were obtained for a concrete breast cancer patient. The accumulation of points for each clinical feature and risk score can predict the survival of breast cancer patients in 1, 3, and 5 years after diagnosis. The discrimination and calibration of the nomogram were validated using C-index and calibration curve. C-index varied from 0.5 to 1. The feasibility of discrimination increased with increasing C-index. The calibration curve of the nomogram in 1, 3, and 5 years was displayed. The closer the calibration curve is to the diagonal line, the more precise the calibration.

### Immune and DNA repair genes in breast cancer

Gene set variation analysis (GSVA) (19) was performed with the single-sample gene set enrichment analysis (ssGSEA) method (20, 21) to calculate the immune infiltration score of 16 immune cells and 13 immune-related pathways. The infiltration scores reflect the activity of immune cells or immune-related pathways. The infiltration scores of the tumor sample in the high- and low-risk groups were counted, respectively, and compared using the Wilcoxon rank-sum test. The immune infiltration scores were presented by a box plot. The annotated gene set file was applied in the ssGSEA analysis (**Supplementary Table 2**).

Considering the density relationship with DNA repair and immune pathway functions in the cancer microenvironment, CIBERSORT was applied to evaluate the immune microenvironment. CIBERSORT (http://cibersort.stanford.edu/) package in R software was invited by Newman et al. (22) according to deconvolution, which can quantify the enrichment of immune cells in many cases. The abundance of 22 kinds of infiltrated immune cells (plasma cells, dendritic cells, CD4^+^ T cells, CD8^+^ T cells, regulatory T cells, natural killer cells, mast cells, naive B cells, memory B cells, and macrophages) was quantified in breast cancer samples. Each of the specimens was estimated based on their gene expression profile retrieved from the TCGA database. Breast cancer patients were divided into low- and high-risk groups according to the expression level of prognosis-related DNA repair genes for CIBERSORT analysis.

The degree of tumor purity and the immunology infiltration level were evaluated using the ESTIMATE algorithm (https://bioinformatics.mdanderson.org/estimate/), which applied gene expression profiles as the signature for stromal and immune score estimation (23). ESTIMATE score was the sum of stromal and immune scores, which revealed the tumor purity and the immunology infiltration level. The FPKM normalized RNA-seq expression profile of breast cancer specimens was downloaded from the TCGA database to perform an ESTIMATE calculation. Breast cancer patients were divided into low- and high-risk groups using the risk score to compare tumor purity and immune infiltration.

### Anticarcinogen sensitivity analysis

In total, 12 types of anticarcinogen (bexarotene, camptothecin, cisplatin, docetaxel, etoposide, gemcitabine, imatinib, methotrexate, paclitaxel, rapamycin, vinorelbine, vorinostat) were analyzed with their half-maximal inhibitory concentration (IC50) in each breast carcinoma sample from the TCGA gene expression level data. Samples from breast cancer patients were divided into high- and low-risk groups based on DNA repair gene expression level for analysis. The R software “pRRophetic” (4) package was utilized to calculate the IC50 of each drug, whose estimation was based on Genomics of Drug Sensitivity in Cancer (GDSC; http://www.cancerrxgene.org/) (5). The half-maximal inhibitory concentration of drugs between the two groups was compared using the Wilcoxon rank-sum test.

### Consensus clustering for DNA repair genes

Breast cancer specimens were clustered into *k* (2–9) groups using the R software “ConsensusClusterPlus” package based on their expression level of prognosis-significant DNA repair genes. An unsupervised clustering method was applied to optimize the number of cluster groups (*k*-value). Principal component analysis (PCA) of the total gene expression matrix for breast cancer was applied to validate the consensus-clustered groups. The survival and clinical features of breast cancer patients in the two clusters were compared utilizing the Kaplan–Meier analysis with log-rank test and *X* ^2^ test, presented using the Kaplan–Meier curve and heatmap.

### Real-time quantitative PCR

The total RNA from each sample was purified using the Easy Fast Tissue/Cell Kit RNA (TIANGEN Biotech Co., Ltd., Beijing, China). Then, RNA was transcribed into complementary DNA (cDNA) utilizing 5×FastKing-RT SuperMix (TIANGEN Biotech Co., Ltd., Beijing, China). RT-qPCR was performed with 2× PerfectStart II Probe qPCR SuperMix (TransGen Biotech, Beijing, China) to detect the relative expression level of DNA repair (DEGs). ABI Prism7500 was employed to perform the RT-qPCR. The expression level of each gene was normalized using endogenous glyceraldehyde-3-phosphate dehydrogenase (GAPDH) with 2^−△△^Ct algorithms. Sangon Biotech (Shanghai, China) provided the primers for each gene. **Supplementary Table 3** shows the sequences of the qPCR primers.

### Cell culture

The breast cancer cell lines MDA-MB-231, MCF-7, and T47D and the normal breast cell line MCF-10a were purchased from the American Type Culture Collection (ATCC). Cells were grown in Dulbecco’s modified Eagle’s medium (DMEM) supplemented with 10% fetal bovine serum (FBS) and 1% penicillin–streptomycin with 5% CO_2_ at 37°C. MCF-10a cell lines were maintained in Ham’s F-12, supplemented with 10% horse serum, insulin (10 μg/ml), epidermal growth factor (20 ng/ml), cholera toxin (100 ng/ml), and hydrocortisone (0.5 μg/ml) with 5% CO_2_ at 37°C.

## Results

### Sorting DEGs and performing biofunctional enrichment analysis

**Figure 1:**
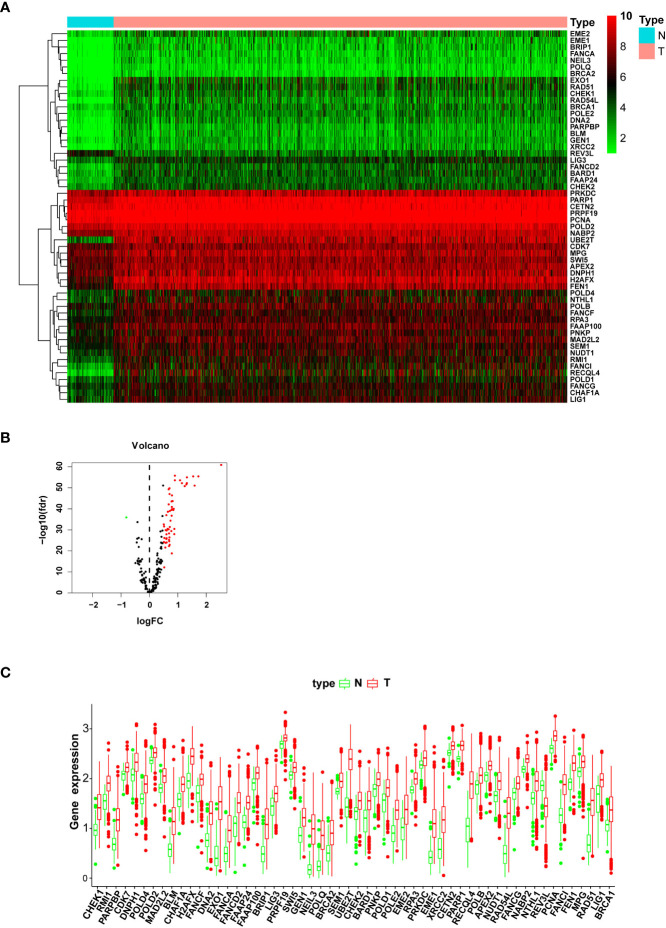
The expression level of differentially expressed genes (DEGs) between the normal and tumor groups. **(A)** The heatmap shows the expression levels of DEGs; downregulated genes are shown in green, and upregulated genes are presented in red. **(B)** The volcano plot presents the expression levels and expression fold changes of DEGs; one downregulated gene is shown as a green dot; 55 unregulated genes are presented as red dots. **(C)** The box bar presents the expression levels of DEGs in normal and tumor tissues.

The gene expression level profiles of 113 normal breast and 1,104 breast cancer specimens were downloaded from the TCGA database. Totally, 56 DEGs were retrieved (**Table 1**). The expression level of 55 genes was upregulated, and one gene was downregulated in the tumor specimens compared with the normal samples. Heatmap and box plot were employed to identify the relative expression level of DEGs. A volcano plot was utilized to indicate the fold change of DEG expression level in the tumor group compared with the normal group (**Figures 1A–C**). In the GO analysis, for the biological process (BP) category, DEGs were mainly enriched in double-strand break repair and DNA replication and recombination. For the cellular components (CC) category, DEGs were mainly enriched in the chromosome (telomeric region), nuclear chromosome, and DNA polymerase complex. For the molecular function (MF) category, DEGs were mainly enriched in catalytic activity acting on DNA, damaged DNA binding, and nuclease activity (**Supplementary Figures S1A–C**). In the KEGG pathway analysis, DEGs were mainly enriched in the base excision repair pathway, homologous recombination, and DNA replication (**Supplementary Figures S1D–F**). These pathways help cancer cells live a better life under DNA damage due to the toxicity of chemotherapy drugs or ionizing radiation therapy.

**Table 1:** The expression level of differentially expressed DNA repair genes between normal breast tissues and breast cancer tissues.

Gene	conMean	treatMean	logFC	*P*-value	FDR
*CHEK1*	0.98877	1.785238	0.796468	1.49E−41	1.29E−40
*RMI1*	1.903181	2.758283	0.855102	1.07E−41	9.75E−41
*PARPBP*	0.638258	1.31578	0.677522	7.57E−44	7.57E−43
*CDK7*	3.163296	3.688101	0.524805	1.88E−27	7.38E−27
*DNPH1*	3.278147	4.065005	0.786859	5.84E−20	1.60E−19
*POLD4*	2.032654	2.736838	0.704183	9.79E−28	4.00E−27
*POLD2*	4.176734	4.776556	0.599822	3.17E−29	1.35E−28
*MAD2L2*	2.533546	3.223759	2.0.690213	1.08E−29	4.79E−29
*BLM*	0.520341	1.298436	0.778095	2.83E−45	2.98E−44
*CHAF1A*	1.970806	2.751902	0.781097	2.60E−38	1.80E−37
*H2AFX*	2.926563	4.517459	1.590896	5.12E−53	8.54E−52
*FANCF*	2.1997	2.941967	0.742266	6.75E−36	4.22E−35
*DNA2*	0.726904	1.54369	0.816786	1.81E−45	2.01E−44
*EXO1*	0.411267	1.951769	1.540502	5.77E−58	3.44E−56
*FANCA*	0.433598	1.081486	0.647887	3.35E−40	2.48E−39
*FANCD2*	1.073689	1.915102	0.841413	4.54E−41	3.63E−40
*FAAP24*	1.185788	1.891448	0.705661	1.12E−51	1.61E−50
*FAAP100*	2.666604	3.341689	0.675085	1.02E−31	5.22E−31
*BRIP1*	0.436727	1.224791	0.788064	1.27E−42	1.21E−41
*LIG3*	1.678692	2.275165	0.596474	5.62E−27	2.08E−26
*PRPF19*	5.398201	6.038054	0.639853	6.47E−32	3.50E−31
*SWI5*	3.199491	3.706935	0.507444	1.79E−25	6.16E−25
*GEN1*	0.859216	1.376169	0.516953	4.17E−33	2.39E−32
*NEIL3*	0.164244	1.061309	0.897066	1.62E−58	1.62E−56
*POLQ*	0.214743	0.890601	0.675859	3.48E−51	4.64E−50
*BRCA2*	0.444422	0.949933	0.50551	4.36E−34	2.56E−33
*SEM1*	2.391233	2.962014	0.570781	2.77E−31	1.38E−30
*UBE2T*	1.724791	4.250674	2.525883	5.87E−64	1.17E−61
*CHEK2*	1.440398	2.008462	0.568064	4.72E−25	1.52E−24
*BARD1*	1.261635	1.977508	0.715873	7.70E−33	4.28E−32
*PNKP*	2.389707	2.996326	0.606618	2.99E−25	9.98E−25
*POLD1*	1.86593	2.562465	0.696535	7.76E−26	2.72E−25
*POLE2*	0.856138	1.648147	0.792009	3.33E−48	3.92E−47
*EME2*	1.004433	1.71542	0.710987	4.30E−24	1.32E−23
*RPA3*	2.432154	3.027863	0.595709	1.41E−38	1.00E−37
*PRKDC*	3.823971	4.343951	0.519981	3.63E−13	7.18E−13
*EME1*	0.365843	1.262187	0.896343	8.87E−56	2.53E−54
*XRCC2*	0.562176	1.334904	0.772728	4.22E−41	3.52E−40
*CETN2*	4.726436	5.334299	0.607863	3.84E−31	1.87E−30
*PARP1*	4.265424	5.342225	1.076801	8.45E−56	2.53E−54
*RECQL4*	1.025358	2.749565	1.724207	6.88E−58	3.44E−56
*POLB*	2.552774	3.243129	0.690355	1.76E−23	5.35E−23
*APEX2*	3.109617	3.825526	0.715909	1.37E−40	1.06E−39
*NUDT1*	2.161085	2.932469	0.771384	7.94E−31	3.69E−30
*RAD54L*	0.433436	1.578687	1.145251	2.63E−54	5.85E−53
*FANCG*	2.201891	2.805572	0.603681	2.58E−27	9.94E−27
*NABP2*	3.570932	4.278414	0.707482	6.95E−49	8.69E−48
*NTHL1*	2.044396	2.919381	0.874986	2.58E−29	1.12E−28
*REV3L*	2.545592	1.732446	−0.81315	1.86E−37	1.20E−36
*PCNA*	5.043073	6.284849	1.241775	1.00E−52	1.54E−51
*FANCI*	1.405207	2.723608	1.318401	2.54E−54	5.85E−53
*FEN1*	2.723662	4.02405	1.300388	1.21E−53	2.42E−52
*MPG*	3.375279	4.065658	0.690379	1.53E−26	5.48E−26
*RAD51*	0.647756	1.967774	1.320018	2.50E−57	1.00E−55
*LIG1*	2.062727	2.940332	0.877605	7.01E−32	3.69E−31
*BRCA1*	1.10724	1.666588	0.559348	8.74E−23	2.53E−22

LogFC, log_2_ (fold change); FDR, false discovery rate.

### Retrieving prognostic-significant DNA repair genes

**Figure 2:**
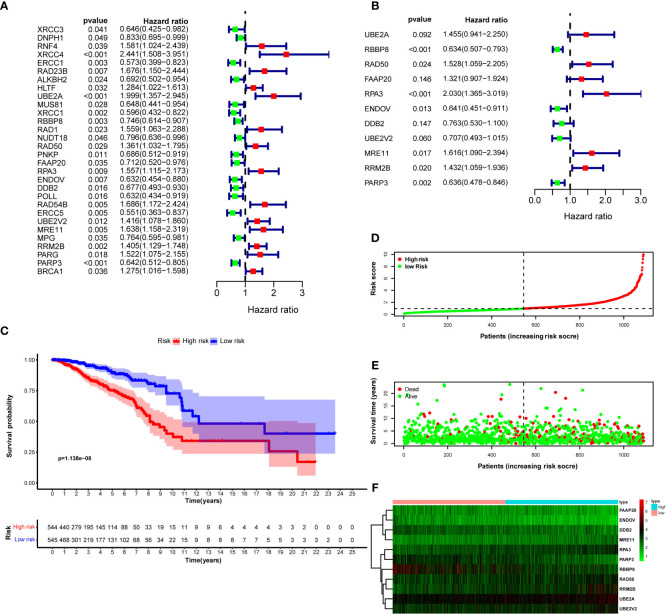
DNA repair genes and Kaplan–Meier curve forest plot of the hazard ratio for the high- and low-risk groups. Breast cancer patients were stratified into high- and low-risk groups using risk scores counted by the expression level of prognosis-related DNA repair genes for analysis. **(A)** Forest plot of 30 prognosis-related DNA repair genes retrieved using univariate Cox regression. **(B)** Forest plot of 11 prognosis-related genes retrieved using multivariate Cox regression model after optimizing by the AIC value. **(C)** KM curve of OS for breast cancer patients in the high- and low-risk groups stratified by the median of risk scores. **(D)** The dotted line displays the individual inflection point of the risk score curve and shows that patients are divided into low- and high-risk groups using median risk score; red dots represent patients with high risk; green dots represent patients with low risk. **(E)** Risk score scatter plot of the high- and low-risk patient groups; red dots show dead patients; green dots show alive patients; the survival time of dead patients decreased with the risk score ascending. **(F)** Risk score heatmap of 11 prognosis-related DNA repair genes; the expression level of 11 DNA repair genes increased with color varied from green to red.

Univariate Cox proportional hazard regression with the Wald *X* ^2^ test identified 30 DNA repair genes (*XRCC3*, *DNPH1*, *RNF4*, *XRCC4* , *ERCC1*, *RAD23B*, *ALKBH2*, *HLTF*, *UBE2A*, *MUS81*, *XRCC1* , *RBBP8*, *RAD1*, *NUDT18*, *RAD50* , *PNKP*, *FAAP20*, *RPA3*, *ENDOV*, *DDB2*, *POLL*, *RAD54B*, *ERCC5*, *UBE2V2*, *MRE11* , *MPG*, *RRM2B*, *PARG*, *PARP3* , and *BRCA1* ) as prognosis indicators for breast cancer (**Figure 2A**). They were involved in constructing a multivariate Cox regression model optimized by the AIC value to avoid overfitting. Finally, we studied 11 DNA repair genes (*UBE2A*, *RBBP8*, *RAD50* , *FAAP20*, *RPA3*, *ENDOV*, *DDB2*, *UBE2V2*, *MRE11* , *RRM2B*, and *PARP3* ) in the multivariate Cox regression model (**Figure 2B**; **Table 2**). The hazard ratio of five genes (*RBBP8*, *PARP3* , *ENDOV*, *UBE2V2*, and *DDB2*) was <1, which plays a protective role in developing breast cancer. The hazard ratio of other six genes (*FAAP20*, *RRM2B*, *UBE2A*, *RAD50* , *MRE11* , and *RPA3*) was >1, regarded as risk factors in developing breast cancer. The coefficient of these 11 genes and their expression level were combined to calculate the risk score for each patient. Breast cancer patients were classified into the high-risk group (*n* = 545) and the low-risk group (*n* = 545) using the median risk score. Kaplan–Meier analysis with log-rank test proved that the overall survival (OS) between patients in the high- and low-risk groups is statistically significant (median time = 12.2 vs. 8.1 years, log-rank *P* < 0.001) (**Figure 2C**).

**Table 2:** Prognosis-related DNA repair genes.

ID	coef	HR	HR.95L	HR.95H	*P*-value
*UBE2A*	0.375195	1.455275	0.94118	2.25018	0.091539
*RBBP8*	−0.45532	0.634247	0.507224	0.79308	6.52E−05
*RAD50*	0.423817	1.527782	1.058641	2.204825	0.023547
*FAAP20*	0.278518	1.32117	0.90733	1.923766	0.1463
*RPA3*	0.707966	2.029859	1.364873	3.018834	0.000472
*ENDOV*	−0.44476	0.640979	0.450874	0.911239	0.01322
*DDB2*	−0.27012	0.76329	0.529641	1.100011	0.147412
*UBE2V2*	−0.34671	0.70701	0.492544	1.014859	0.06011
*MRE11*	0.47965	1.615509	1.090373	2.393557	0.016788
*RRM2B*	0.358795	1.431603	1.058847	1.935585	0.019725
*PARP3*	−0.45253	0.636017	0.478138	0.846027	0.00188

Eleven DNA repair genes were related to the overall survival of breast cancer patients and applied to calculate the risk scores which stratify the patients into high - and low-risk groups.

### Prognostic hazard curves and heatmap

To evaluate the difference in the survival of breast cancer patients between the two groups and their relationship with risk score, the risk curves for breast cancer patients were drawn to visualize the risk score for each breast cancer patient. The relationship between risk score and survival duration after breast cancer diagnosis was displayed by a scatter plot (**Figures 2D, E**). These results revealed that patients with higher risk scores live shorter after a breast cancer diagnosis. The risk heatmap was drawn to validate the expression level of significant prognostic genes in the tumor tissues of high- and low-risk patients. According to the risk heatmap, *RBBP8* was downregulated in the high-risk group, indicating its tumor suppression potential in breast cancer, and *UBE2A* acted as a tumor-accelerating gene because it was upregulated in the high-risk group (**Figure 2F**).

## Screening prognostic factors and constructing a predictive model for OS of breast cancer patients

**Figure 3:**
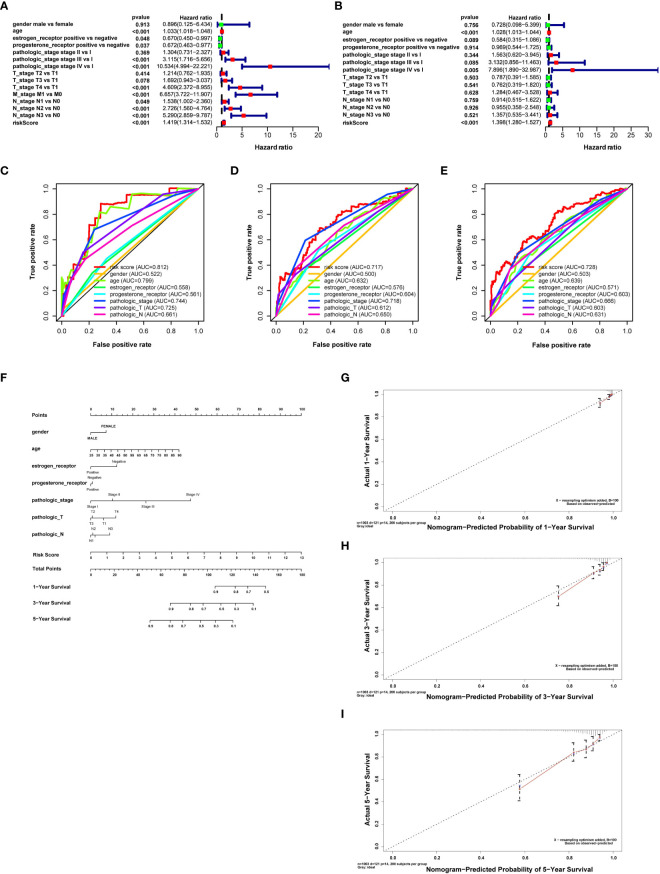
Retrieving survival indicators, evaluating their discrimination, and constructing a prognostic nomogram. **(A)** Forest plot for risk score and clinical features in the univariate Cox proportional risk regression model. **(B)** Forest plot for risk score and clinical features in the multivariate Cox proportional risk regression model. **(C)** Receiver operating characteristic (ROC) curves for evaluating the discrimination of survival indicators in 1 year. **(D)** ROC curves to evaluate the discrimination of survival indicators in 3 years. **(E)** ROC curves for evaluating the discrimination of survival indicators in 5 years. The risk score has better prognosis discrimination feasibility than other clinical features; the discrimination feasibility increased with the ascending of AUC. **(F)** Nomogram for breast cancer with gender, age, clinical stage, T and N stages, estrogen receptor, progesterone receptor, and risk score calculated by prognosis-related DNA repair genes predicting survival in 1, 3, and 5 years. **(G)** Calibration curves of the prognostic nomogram prediction in 1 year. **(H)** Calibration curves of the prognostic nomogram prediction in 3 years. **(I)** Calibration curves of the prognostic nomogram prediction in 5 years.

Univariate Cox regression analysis was performed with a risk score and other clinical factors to investigate their prognosis prediction value. Age [hazard ratio (HR) = 1.033, *P* < 0.001], estrogen receptor (HR = 0.670, *P* = 0.048), progesterone receptor (HR = 0.672, *P* = 0.037), pathologic stage (stage III vs. stage I, HR = 3.115, *P* < 0.001; stage IV vs. stage I, HR = 10.534, *P* < 0.001), T stage (T4 vs. T1, HR = 4.608, *P* < 0.001), M stage (M1 vs. M0, HR = 6.657, *P* < 0.001), N stage (N1 vs. N0, HR = 1.538, *P* = 0.048; N2 vs. N0, HR = 2.725, *P* < 0.001; N3 vs. N0, HR = 5.290, *P* < 0.001), and risk score (HR = 1.418, *P* < 0.001) were correlated with OS of breast cancer patients (**Figure 3A**). Multivariate Cox regression analysis of these clinical features showed that age (HR = 1.028, *P* < 0.001), pathologic stage (stage IV vs. stage I, HR = 7.895, *P* = 0.004), and risk score (HR = 1.397, *P* < 0.001) were independent risk factors for survival (**Figure 3B**).

Moreover, we assessed the feasibility of each prognostic predictor to discriminate between alive or dead patients using the AUC values in the ROC curve. The AUC of risk score was more significant than age, stage, and T stage in both 1 and 5 years. The AUC of risk score was similar to T stage in 3 years and larger than any other clinical features, indicating that risk score was a better prognostic predictor than other clinical features (risk score AUC = 0.812, 0.717, and 0.728 for 1, 3, and 5 years, respectively) (**Figures 3C–E**).

A nomogram was drawn to display the constructed prognostic model for breast cancer patients. Gender, age, estrogen receptor, progesterone receptor, tumor stage, T and N stages, and risk score were selected to establish the nomogram (**Figure 3F**). Results revealed that the C-index of the constructed nomogram was 0.810. **Figures 3G–I** display the calibration curve of the nomogram in 1, 3, and 5 years. The C-index and calibration curve demonstrated that the nomogram could partially predict the prognosis of breast cancer patients.

### Correlation analysis between clinical features and DNA repair-related gene predictor

According to the number of categories of clinical features, we applied a *t*-test or Kruskal–Wallis test to evaluate the correlations between the risk score or expression level of 11 prognostic DNA repair genes and clinical features. The results showed that the expression level of *PARP3* , *ENDOV*, and *UBE2A* was distributed distinctively between men and women (*P* = 0.031, 0.021, 0.004, **Supplementary Figure S2A**). The expression level of *ENDOV*, *FAAP20*, *MRE11* , *PARP3* , *RAD50* , *RBBP8*, *RPA3*, *RRM2B*, *UBE2A*, and *UBE2V2* and the risk scores were distributed distinctively between the different races (*P* = 0.002, 1.653e−11, 0.009, 0.046, 1.296e−19, 1.921e−05, 2.942e−06, 4.286e−09, 7.451e−05, 0.011, 0.021) (**Supplementary Figure S2B**). The expression level of *RRM2B*, *RPA3*, *PARP3* , and *RAD50* was increased with the ascending age of patients (**Supplementary Figure S2C**). The expression level of *RBBP8* decreased with the increasing age of patients. In estrogen receptor-positive breast cancer patients, the expression level of *RRM2B*, *RPA3*, *RBBP8*, *RAD50* , *PARP3* , *ENDOV*, *DDB2*, and *FAAP20* was higher than in estrogen-negative breast cancer patients. In estrogen receptor-negative breast cancer patients, the expression level of *UBE2V2*, *UBE2A*, and *MRE11* was higher than in estrogen-positive patients (**Supplementary Figure S2D**). In progesterone receptor-positive breast cancer patients, the expression level of *PARP3* , *RAD50* , *ENDOV*, *FAAP20*, *RRM2B*, *DDB2*, *RBBP8*, and *RPA3* was higher than in progesterone receptor-negative breast cancer patients. In progesterone receptor-negative breast cancer patients, the expression level of *UBE2V2* and *UBE2A* was higher than in patients with progesterone receptor-positive breast cancer. Furthermore, the risk score was higher in patients with progesterone receptor-negative breast cancer than in positive patients, which might indicate that PR-negative breast cancer leads to a higher risk of death in patients (**Supplementary Figure S2E**). The expression level of *PARP3* , *RBBP8*, and *DDB2* was higher in breast cancer patients without *HER2* receptors than in patients positive for the *HER2* receptor. The risk score was higher in *HER2*-positive patients (**Supplementary Figure S2F**). The expression level decreased with advanced clinical stage (*P* = 7.397e−04 and 8.969e−04, respectively). The protective role of *RBBP8* and *PARP3* in developing breast cancer, implied by utilizing multivariate Cox regression, was validated. The risk score was higher in advanced pathologic stage patients than in elementary pathological stage patients (*P* = 8.231e−05), implying that the advanced pathology stage plays a dangerous role in breast cancer patients with the development of disease (**Supplementary Figure S2G**). The expression level of *RBBP8* decreased with ascending T stage, implying its protective role for the breast cancer patient. The expression level of *FAAP20* was increased with ascending T stage, indicating its important role in tumor growth. The risk score was increased with the ascending of T stage. It means that patients with a lower T stage have a better prognosis (**Supplementary Figure S2H**). The expression level of *RBBP8* was lower in M1-stage breast cancer patients than in M0-stage patients. Accordingly, *RBBP8* may prevent breast cancer cells from organ metastasis (**Supplementary Figure S2I**). The expression level of *DDB2*, *RPA3*, *RAD50* , *RRM2B*, and *UBE2A* was higher in breast cancer tissues with lymph node metastasis compared with breast cancer tissues without lymph node metastasis, which was estimated by the distribution of their expression level between N0 and N1–N3 stages. Similarly, the risk score increased with a higher N stage (**Supplementary Figure S2J**).

### External validation of risk score

**Figure 4:**
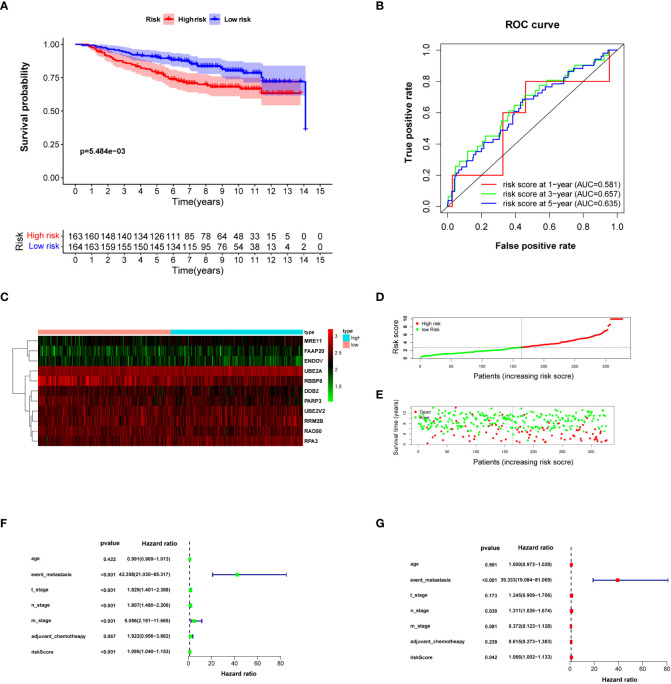
Validating risk score calculated by prognosis-significant DNA repair genes in GSE20685. **(A)** Kaplan–Meier analysis of patients from the high- and low-risk groups. **(B)** ROC curve of the risk score in 1, 3, and 5 years. **(C)** Risk score heatmap of 10 prognosis-related DNA repair genes; the expression level of eight DEGs increased with color varied from green to red. **(D)** Risk score scatter plot of the high-risk and low-risk patient groups. **(E)** The dotted line displays the individual inflection point of the risk score curve. **(F, G)** Forest plot of univariate and multivariate Cox regression for prognosis indicators including risk score.

The risk score for each breast cancer patient in the validation set was estimated according to a formula constructed by the data downloaded from the TCGA database and the expression level of prognostic DNA repair genes in the GSE20685 dataset. The patients in GSE20685 were divided into high- and low-risk groups according to the median risk score. The difference in OS between the high- and low-risk groups was statistically significant (**Figure 4A**) (log-rank test *P* = 5.484e−03). The AUC of the ROC curve estimated the feasibility of prognosis prediction for risk score time-dependent at 1, 2, and 3 years (**Figure 4B**). Risk curves and prognosis hazard heatmaps were drawn to analyze the risk role of sorted prognostic DNA repair genes (**Figures 4C–E**). Univariate and multivariate Cox regression revealed that risk score is an independent prognosis indicator for breast cancer patients (**Figures 4F, G**). A similar result could be drawn for the GSE20685 cohort compared with the TCGA breast cancer cohort.

### Analysis of the immune filtration score and anticarcinogen sensitivity

**Figure 5:**
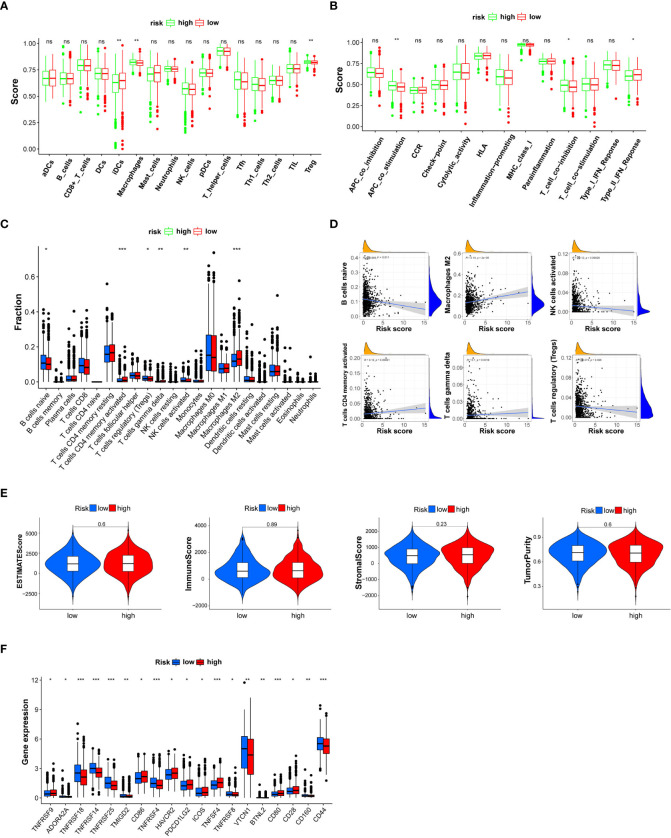
Box plot for the single-sample gene set enrichment analysis (ssGSEA) immune score, CIBERSORT immune cell fraction, and ESTIMATE immune filtration analysis between patients in the high- and low-risk groups categorized by a median risk score. **(A)** The scores of 16 immune cells are estimated by ssGSEA. **(B)** The scores of 13 immune-related functions are calculated by ssGSEA. **(C)** The fraction of each immune cell is estimated by CIBERSORT. **(D)** Linear correlation analysis between the fraction of each immune cell and risk score. **(E)** ESTIMATE score, immune score, stromal score, and tumor purity. **(F)** The expression level of immune checkpoint genes is statistically different between the high- and low-risk breast cancer groups. NK, natural killer; DCs, dendritic cells; iDCs, immature DCs; pDCs, plasmacytoid dendritic cells; TIL, tumor-infiltrating lymphocyte; CCR, cytokine–cytokine receptor; APC, antigen-presenting cell. Adjusted *P*-values are shown as ns (not significant), *P < 0.05, **P < 0.01, ***P < 0.001.

Based on the “ssGSEA” method, included in the R package “GSVA,” the enrichment scores of 16 immune cell subgroups and 13 immune functions were estimated. Two kinds of immune cell subpopulations (macrophages and Tregs) showed higher scores in the high-risk group than in the low-risk group. IDC immune cells showed a higher score in the low-risk group than in the high-risk group (**Figure 5A**). Furthermore, the immune functions of two types (APC cosimulation and T-cell co-inhibition) revealed a higher score in the high-risk group. Type II IFN responses were higher in the low-risk group (**Figure 5B**). CIBERSORT analysis indicated that risk score was positively correlated with activated CD4 memory T cells, gamma delta, and macrophages M2. Nonetheless, the risk score was negatively correlated with B cells naive, regulatory T cells, and NK cells activated (**Figures 5C, D**). The ESTIMATE evaluation explained that the distinction of ESTIMATE score, immune score, stromal score, and tumor purity between the high- and low-risk groups was statistically insignificant (*P* > 0.05) (**Figure 5E**). The expression level of immune checkpoint genes was expressed differently between breast cancer patients in the high- and low-risk groups (**Figure 5F**). The expression level of *ADORA2A*, *TNFRSF18*, *TNFRSF14*, *TNFRSF25*, *TMIGD2*, *TNFRSF4*, *TNFRSF8*, *VTCN1*, *BTNL2*, *CD160*, and *CD44* was higher in the low-risk group. However, the expression level of *TNFRSF9*, *CD86*, *HAVCR2*, *PDCD1LG2*, *ICOS*, *TNFSF4*, *CD80*, and *CD28* was higher in the high-risk group. These results indicated the response to immune checkpoint inhibitor treatment.

Anticarcinogen sensitivity analysis demonstrated that vinorelbine, rapamycin, paclitaxel, gemcitabine, imatinib, bexarotene, docetaxel, etoposide, methotrexate, and camptothecin were equipped with higher IC50 levels in the high-risk group. This analysis revealed that patients in the low-risk group could be more sensitive to these anticarcinogens (**Supplementary Figures S3A–L**).

### Breast cancer molecular subgroup divided by DNA repair genes

**Figure 6:**
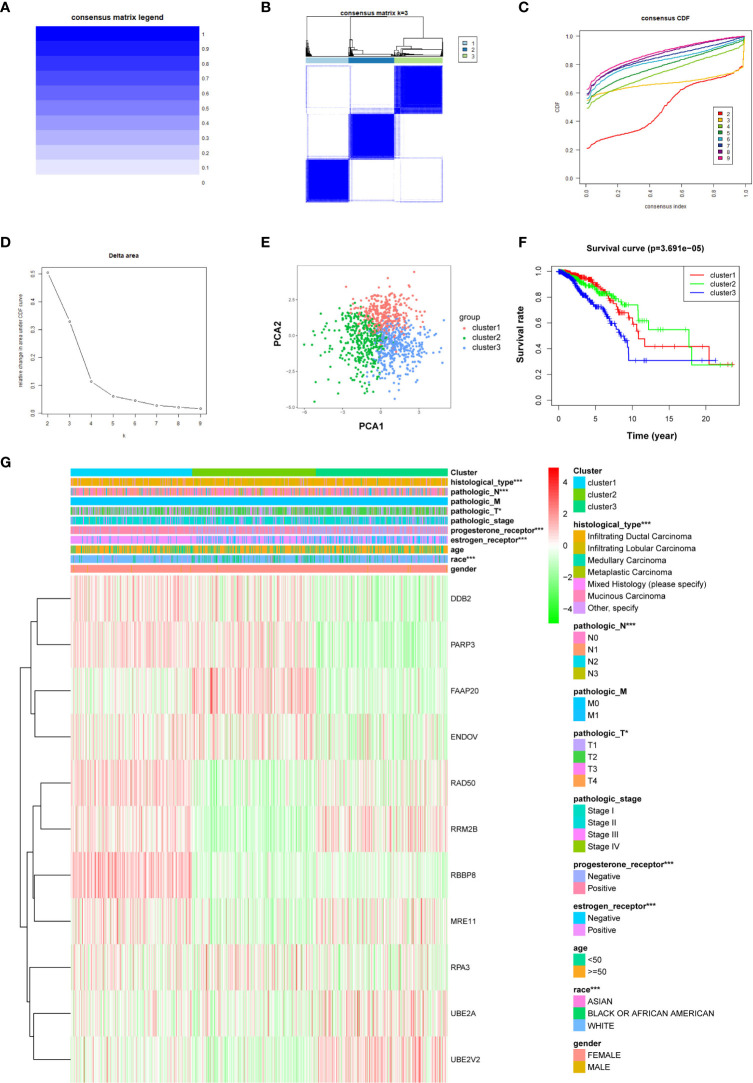
The R “ConsensusClusterPlus” package is applied to stratify breast cancer samples into three clusters equipped with different prognoses. **(A)** Consensus matrix legend. **(B)** Consensus clustering matrix for *k* = 3. **(C)** Consensus clustering cumulative distribution function (CDF) for *k* = 2–9. **(D)** Relative change in AUC of CDF. **(E)** PCA of the expression profiles of DNA repair genes from clusters 1, 2, and 3. **(F)** Kaplan–Meier curve of OS of patients between clusters 1, 2, and 3. **(G)** Heatmap of clinical features and three clusters of breast cancer patients. *P < 0.05, ***P < 0.001.

To investigate the characteristics of DNA repair genes in breast cancer, we divided the TCGA breast cancer samples into different subgroups depending on the expression similarity of 11 DNA repair-related genes utilizing the R package software “ConsensusClusterPlus.” Based on the expression similarity of DNA repair-related genes, *k* = 3 appeared to be an adequate choice, with clustering stability rising from *k* = 2 to 9 for the TCGA datasets. The subgroups were named cluster 1, cluster 2, and cluster 3 (**Figures 6A–D**). The PCA analysis indicated that the total gene expression matrix could be validated by the consensus cluster of breast cancer samples by the expression level of prognostic DNA repair genes (**Figure 6E**). The Kaplan–Meier analysis showed that the OS of breast cancer patients in cluster 3 was the lowest among the three clusters (**Figure 6F**) (*P* = 3.691e−05). Further analysis revealed that histology types, N and T stages, progesterone receptor, estrogen receptor, and age differed significantly between these three subgroups (**Figure 6G**).

## Expression levels of DEGs in breast cancer tissues and cell lines

**Figure 7:**
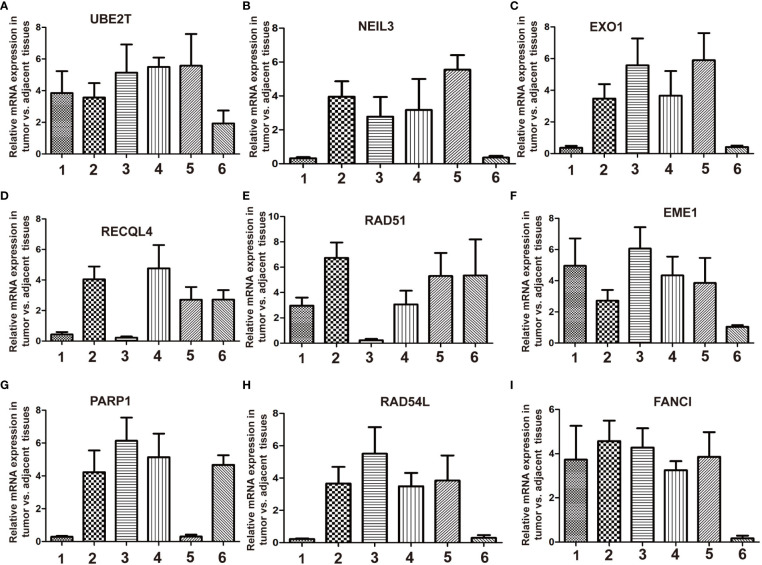
RT-qPCR validation of the relative expression level of DEGs between breast cancer and adjacent normal tissues. Total RNA is isolated from six pairs of clinical breast cancer and adjacent normal tissues. Relative mRNA expression was analyzed by qPCR. **(A)** *UBE2T*, **(B)** *NEIL3*, **(C)** *EXO1*, **(D)** *RECQL4*, **(E)** *RAD51*, **(F)** *EME1*, **(G)** *PARP1*, **(H)** *RAD54L*, and **(I)** *FANCI*.

To verify the sorted DEGs between breast cancer samples and normal breast tissues, RT-qPCR was performed on six breast cancer specimens with corresponding para-carcinoma tissues. The RT-qPCR results showed that the expression levels of nine DEGs, namely, *UBE2T*, *NEIL3*, *EXO1*, *RECQL4*, *RAD51*, *EME1*, *PARP1*, *RAD54L*, and *FANCI*, retrieved from the TCGA database, were upregulated in the tumor group compared with the adjacent normal tissues (**Figures 7A–I**). The breast cancer cell lines (MCF7, T47D, and MDA-MB-231) showed the same results as the normal breast cell line MCF-10a (**Supplementary Figures S4A–I**). These results revealed that DNA repair was strengthened when normal tissues were translated into tumor tissues. These genes should be validated in larger-scale clinical studies in the future. The molecular biological function of these genes deserves further exploration.

## Discussion

Cells store vital genetic information in DNA whose integrity and stability impact their viability. DNA damage factors, including chemical substances and iron radiation, might produce fatal effects on cells. Many DDR mechanisms have been developed to defend against these factors (24–26). Malignant transformation of cells is usually accompanied by genome instability, which leads to the accumulation of mutations in genetic material. The most famous mutation is *BRCA1* /2, whose upstream or downstream gene mutation leads to the “BRCAness” phenotype (13, 27). In normal cells, DNA repair prevents malignant transformation (7). However, with the origin of the tumor and its expansion or metastasis, DDR becomes the mechanism of chemoresistance and radioresistance for cancer cells, which can lead to tumor relapse after treatment (28, 29). Compared with cells in normal tissues, some DDR pathways may have become inactive, making the left DDR pathway more active (29). Synthetic lethality is a widely accepted conception obtained from this phenomenon, which showed that inhibition of one active DNA repair pathway kills the efficiency of cancer cells when another DNA repair pathway has become inactive (30–32). Herein, we screened 11 breast cancer-related DNA repair genes that could efficiently predict breast cancer prognosis. Furthermore, a functional analysis of immune cells and immune pathways was performed according to the groups stratified by this model. The genes sorted could be potential diagnostic markers or treatment targets for further research.

We retrieved the breast cancer dataset from the TCGA RNA-seq database and found that 55 DNA repair genes were unregulated, while only one was downregulated in breast cancer compared with normal breast tissues. A total of 11 DNA repair genes, namely, *UBE2A*, *RBBP8*, *RAD50* , *FAAP20*, *RPA3*, *ENDOV*, *DDB2*, *UBE2V2*, *MRE11* , *RRM2B*, and *PARP3* , were involved in the prognostic model for breast cancer patients. The risk score for breast cancer patients proved to be valuable in determining the feasibility of survival distinction. We visualized and validated the prognostic model as a nomogram utilizing the C-index and the calibration curve.

The PARP family includes 17 members and involves many biofunctions, including DNA repair, apoptosis, histone binding, and synthetic lethality. Presently, PARPi mainly concentrates on parp1 protein, a nuclear enzyme. When a single-strand break occurs in DNA, PARP1 is activated, and the broken wound on the DNA strand is recognized. Then, *XRCC1*, *POLβ*, and DNA ligase III are recruited into the broken wound, after which the SSB is repaired. Some parp1 inhibitors, including rucaparib and niraparib, are successful PARP-targeted treatments (33, 34). Parp1 is responsible for more than 90% of the work for DNA SSB repair (35). Herein, we discovered that *PARP3* expression plays a protective role in the development of breast cancer (HR < 1). The role of *PARP3* in DNA repair remains unclear. *PARP3* can recruit the aprataxin-like factor (*APLF*) to the DNA damage site (36). The interaction between *APLF* and *PARP3* accelerates the recruitment or retention of *XRCC4*/DNA ligIV at the DNA break site, which promotes the efficiency of DSB repair. Therefore, the biofunction of *PARP3* in the progression of breast cancer deserves further research.

*RAD50* and *MRE11* participate in forming the MRN (*MRE11*-*RAD50*-NBS1) complex, playing an essential role in DDR (37). The MRN complexes worked as sensors and responders for DNA damage, repairing DSBs, replication fork (RF) collapse, dysfunction of telomeres, and virus infection (38). The initiation of DNA repair is usually accompanied by a cell cycle halt. The MRN complex activates ATM and ATR proteins which trigger cell cycle checkpoint response and play a key role in subsequent DNA repair pathways (30, 39, 40). Homologous recombination (HR) and NHEJ are the two main pathways to repair DSB, and they compete with each other when DSB repair is activated. The activity of the MRN complex is prone to choose the HR pathway using the *MRE11* endonuclease cut, which could inhibit NHEJ by producing 3′ ssDNA overhangs. Then, HR was triggered by *MRE11* exonuclease and *EXO1/BLM* bidirectional resection (41). The collapse of the replication fork is another reason for DNA damage, which ATR primarily regulates (42). The MRN complex plays a dual role in treating RF collapse. It could bind to the stalled RF, mediate ATR activation, and promote HR initiation (43–45).

Although the MRN complex may lead to fork degradation (46), knocking out an arbitrary component of MRN in mice is fatal to embryos (47–49). Similarly, mutation of any component of MRN leads to genome instability and is the origin of many diseases, including ataxia-telangiectasia-like disorder (ATLD) and Nijmegen break syndrome (50). As a component of the MRN complex, *RAD50* mutates in acute myeloid leukemia (51), Burkitt lymphoma (52), and endometrial carcinoma (53).

Many studies have focused on the mechanism of DDR in both normal and cancer cells. Some creative DNA repair gene-targeted treatments have been invited to benefit breast cancer patients. We excavated the TCGA database to provide a list of DNA repair genes related to prognosis to predict prognosis in breast cancer patients. This prognostic model may contribute to the tertiary prevention of breast cancer. We hope that our study can provide physicians and scientists with a new horizon for breast cancer research.

This study has some shortcomings. First, the number of patients is limited, which might affect the precision of the prognostic model. A larger cohort of breast cancer patients could be involved in the construction of a better prognostic model. Second, Cox regression is a conventional method for clinical research that has been widely applied for 10 years. Artificial intelligence (AI) has been involved in medical applications nowadays. Combining the prognostic model and AI could provide us with a more precise survival prediction in the future. Third, we should have estimated the degree of deficiency of DNA repair based on the expression level of the DNA repair gene. DNA repair pathways may be strengthened or impaired in tumor cells, which could be a valuable potential indicator for survival prediction.

## Conclusions

DEGs between breast cancer and normal breast samples were investigated and validated using bioinformatic analysis and qPCR experiments. Eleven prognosis-related DNA repair gene signatures were retrieved, which could construct a novel survival prediction model and divide breast cancer patients into high- and low-risk groups. The different survival rates between the two groups were statistically significant. Immune analysis and anticancer drug sensitivity analysis were performed between the two groups. Finally, the tumor subgroups were clustered using the expression level of prognostic DNA repair genes.

## Data availability statement

The original contributions presented in the study are included in the article/**Supplementary Material**. Further inquiries can be directed to the corresponding authors.

## Ethics statement

The studies involving human participants were reviewed and approved by the Ethics Committee of The Shanghai East Hospital of Tongji University. The patients/participants provided their written informed consent to participate in this study.

## Author contributions

YC conceived the idea for this study and downloaded the data from the database and contributed to real-time quantitative PCR. ZH performed the statistical analysis. HQ, HL, and SY participated in the collection of samples. YS provided experimental technology. JY and CW prepared the figures and wrote the article. All authors approved the final version of the manuscript.

## Supplementary material

Supplementary Table 1 Clinicalpathological data of patients from TCGA database.

Supplementary Table 2 The annotation of 16 immune cells and 13 functions in ssGSEA.

Supplementary Table 3 The sequences of qPCR primers.

Supplementary Figure 1 Differentially expressed DNA repair genes are applied in GO and KEGG enrichment analysis. **(A, D)** GO and KEGG enrichment analysis were shown in bar plot respectively. The significant degree of enrichment was measured by length of bar and depth of color. **(B, E)** GO and KEGG pathway enrichment analyses were presented in circle images respectively. The inside of circle represents the Z-score. The red color represented that the increased expression of genes contributed to significant enrichment primarily. The blue color represented that the decreased expression of gene contributed to significant enrichment primarily. The outer circle standed for various pathways, in which the down-regulated genes presented as blue dots and the up-regulated genes presented as red dots. **(C, F)** GO and KEGG enrichment results were presented in heatmaps respectively. The up-regulated genes were presented in red color. The down-regulated genes were presented in blue color.

Supplementary Figure 2 Box plot for displaying relationship between prognostic DNA repair genes or risk score and clinical features. **(A)** Gender; **(B)** Race; **(C)** Age;**(D)** Estrogen receptor; **(E)** Progesterone receptor; **(F)** *HER2* receptor; **(G)** Stage; **(H)** T stage; **(I)** M stage; **(J)** N stage.

Supplementary Figure 3 The evaluated half maximal inhibitory concentration (IC50) for each of 16 anti-cancer drugs between high-risk group and low risk group were displayed in box plots. **(A)** Bexarotene; **(B)** Camptothecin; **(C)** Cisplatin; **(D)** Docetaxel; **(E)** Etoposide; **(F)** Gemcitabine;**(G)** Imatinib; **(H)** Methotrexate; **(I)** Paclitaxel; **(J)** Rapamycin; **(K)** Vinorelbine; **(L)** Vorinostat; Each dot stands for the estimated IC50 value of corresponding drug in breast cancer sample. The higher IC50 is the less sensitive breast cancer is to this drug.

Supplementary Figure 4 RT-qPCR validation of relative expression level of differentially expressed DNA repair genes between breast cancer cell lines and normal breast cell line. Total RNA was isolated from breast cancer cell lines (MCF7, T47D and MDA-MB-231) and normal breast cell line (MCF-10a). Relative mRNA expression was analyzed by qPCR. **(A)** UBE2T; **(B)** NEIL3; **(C)** EXO1;**(D)** RECQL4; **(E)** RAD51; **(F)** EME1; **(G)** PARP1; **(H)** RAD54L; **(I)** FANCI.

## Glossary

**Table:**

PARPi	poly ADP-ribose polymerase inhibitor
AIC	Akaike information criterion value
UBE2A	ubiquitin conjugating enzyme E2 A
RBBP8	retinoblastoma-binding protein 8
FAAP20	Fanconi anemia core complex-associated protein 20
RPA3	replication factor A protein 3
ENDOV	endonuclease V
DDB2	damage-specific DNA binding protein 2
UBE2V2	ubiquitin-conjugating enzyme E2 variant 2
*MRE11*	Meiotic recombination 11 homolog A
RRM2B	ribonucleotide reductase M2 B
*BRCA1*	breast cancer 1
*BRCA2*	breast cancer 2
PD-1	programmed death 1
PD-L1	programmed cell death-ligand 1
*HER2*	human epidermal growth factor receptor-2
PCA	principal component analysis
XRCC3	X-ray repair complementing defective repair in Chinese hamster cells 3
DNPH1	2′- deoxynucleoside 5′-phosphate N-hydrolase 1
RNF4	ring finger protein 4
*XRCC4*	X-ray repair complementing defective repair in Chinese hamster cells 4
ERCC1	excision repair cross-complementing 1
RAD23B	RAD23 homolog B
ALKBH2	AlkB homolog 2
HLTF	helicase-like transcription factor
UBE2A	recombinant ubiquitin conjugating enzyme E2A
*XRCC1*	Xray repair complementing defective repair in Chinese hamster cells 1
RBBP8	retinoblastoma binding protein 8
NUDT18	nudix hydrolase 18
PNKP	polynucleotide kinase 3′-phosphatase
FAAP20	Fanconi anemia core complex-associated protein 20
RPA3	replication protein A3
DDB2	damage-specific DNA binding protein 2
POLL	DNA polymerase lambda
RAD54B	RAD54 homolog B
ERCC5	excision repair cross-complementing rodent repair deficiency complementation group 5
UBE2V2	ubiquitinconjugating enzyme E2 variant 2
MPG	N-methylpurine DNA glycosylase
RRM2B	ribonucleoside-diphosphate reductase subunit M2 B
PARG	poly ADP-ribose glycohydrolase
*PARP3*	poly ADP-ribose polymerase 3
FAAP20	Fanconi anemia core complex-associated protein 20
RPA3	replication protein A3
PR	progesterone receptor
IDC	interdigitating cell
IFN	interferon
NK	natural killer
ADORA2A	adenosine A2a receptor
TNFRSF18	TNF receptor superfamily member 18
TNFRSF14	TNF receptor superfamily member 14
TNFRSF25	TNF receptor superfamily member 25
TMIGD2	transmembrane and immunoglobulin domain containing 2
TNFRSF4	TNF receptor superfamily member 4
TNFRSF8	TNF receptor superfamily member 8
VTCN1	V-Set domain containing T-cell activation inhibitor 1
BTNL2	butyrophilin-like 2
TNFRSF9	TNF receptor superfamily member 9
HAVCR2	hepatitis a virus cellular receptor 2
PDCD1LG2	programmed cell death 1 ligand 2
ICOS	inducible T-cell costimulator
TNFSF4	TNF superfamily member 4
UBE2T	ubiquitin conjugating enzyme E2 T
NEIL3	Nei-like DNA glycosylase 3
EXO1	exonuclease 1
RECQL4	RecQ-like helicase 4
EME1	essential meiotic structure-specific endonuclease 1
PARP1	poly ADP-ribose polymerase 1
RAD54L	RAD54-like
FANCI	FA complementation group I
ATM	ataxia-telangiectasiamutated gene
ATR	ataxia-telangiectasia and rad-3-related protein
BLM	BLM RecQ-like helicase
SSB	single-strand break
DSB	double-strand break
BER	base excision repair
NER	nucleotide excision repair
MMR	mismatch repair
HRR	homologous recombination repair
NHEJ	non-homologous end joining
DDR	DNA damage repair
FPKM	fragments per kilobase of exon model per million mapped fragments
DEGs	differentially expressed DNA repair genes
FDR	false discovery rate
GO	Gene Ontology
KEGG	Kyoto Encyclopedia of Genes and Genomes
ROC	receiver operating characteristic
AUC	area under the curve
GSVA	gene set variation analysis
ssGSEA	single-sample gene set enrichment analysis
IC50	inhibitory concentration
MF	molecular function
OS	overall survival
*APLF*	aprataxin-like factor
RF	replication fork
ATLD	ataxia-telangiectasia-like disorder
AI	artificial intelligence
