# Supplementary figures and images for: Construction of a DNA damage repair gene signature for predicting prognosis and immune response in breast cancer
Source: Front Oncol. 2023 Jan 11;12:1085632. doi: 10.3389/fonc.2022.1085632 (PMC9875088; doi:10.3389/fonc.2022.1085632)

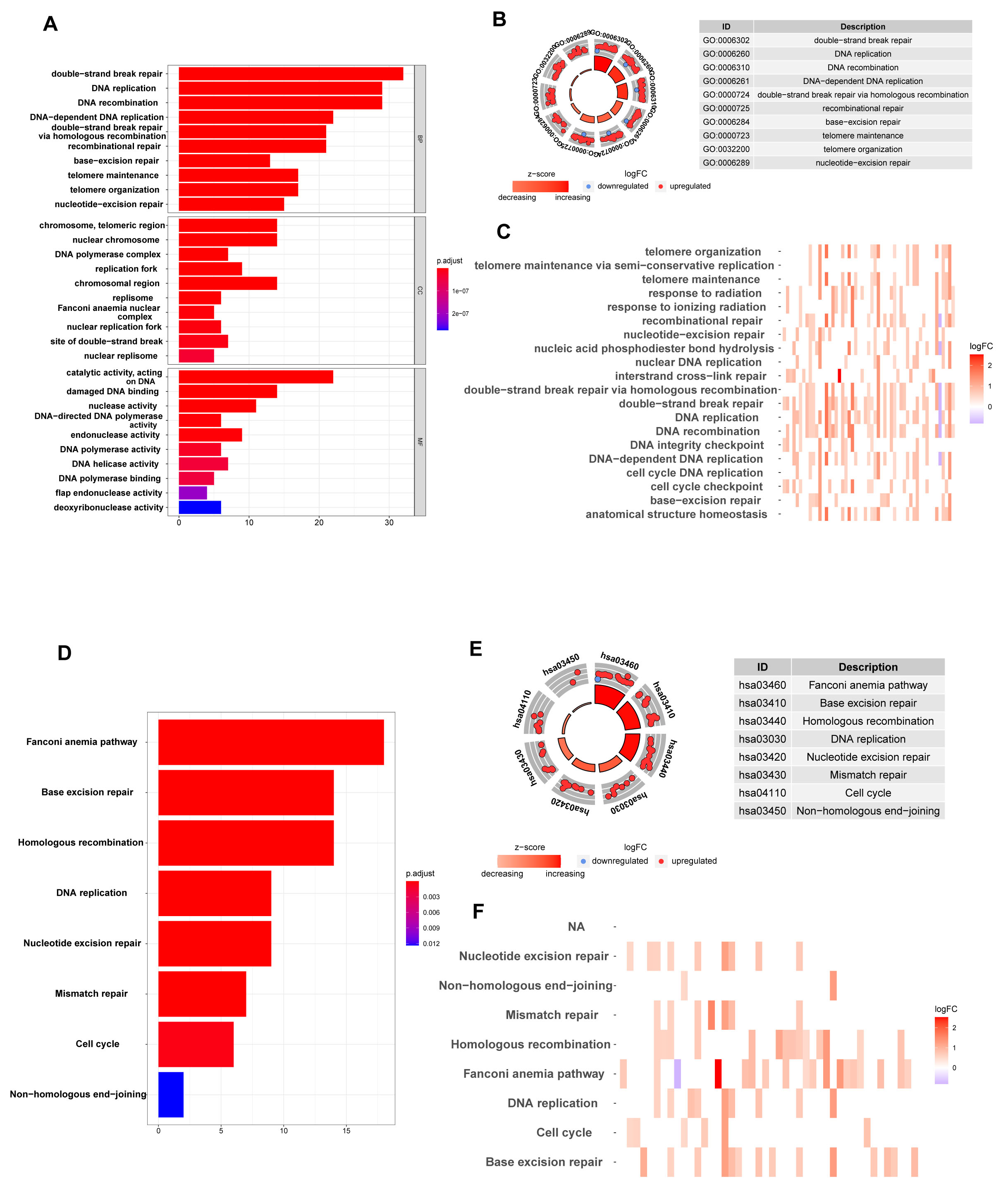

Supplement: Supplementary Figure 1 — Differentially expressed DNA repair genes are applied in GO and KEGG enrichment analysis. (A, D) GO and KEGG enrichment analysis were shown in bar plot respectively. The significant degree of enrichment was measured by length of bar and depth of color. (B, E) GO and KEGG pathway enrichment analyses were presented in circle images respectively. The inside of circle represents the Z-score. The red color represented that the increased expression of genes contributed to significant enrichment primarily. The blue color represented that the decreased expression of gene contributed to significant enrichment primarily. The outer circle standed for various pathways, in which the down-regulated genes presented as blue dots and the up-regulated genes presented as red dots. (C, F) GO and KEGG enrichment results were presented in heatmaps respectively. The up-regulated genes were presented in red color. The down-regulated genes were presented in blue color. [file Image_1.jpeg]

**A**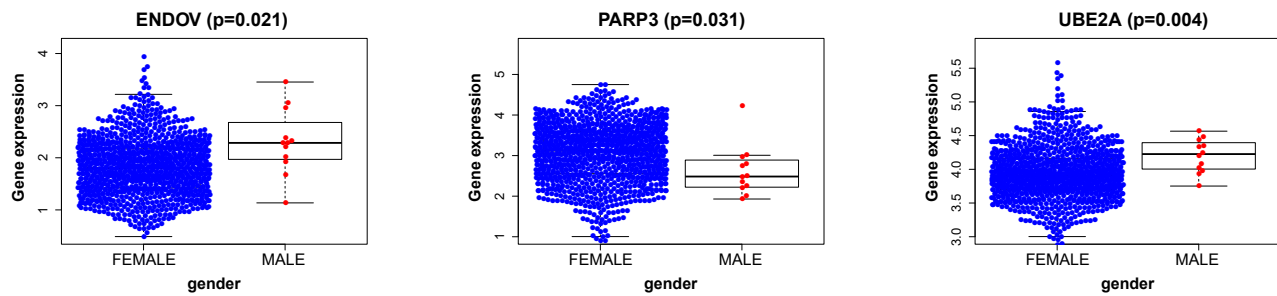**B**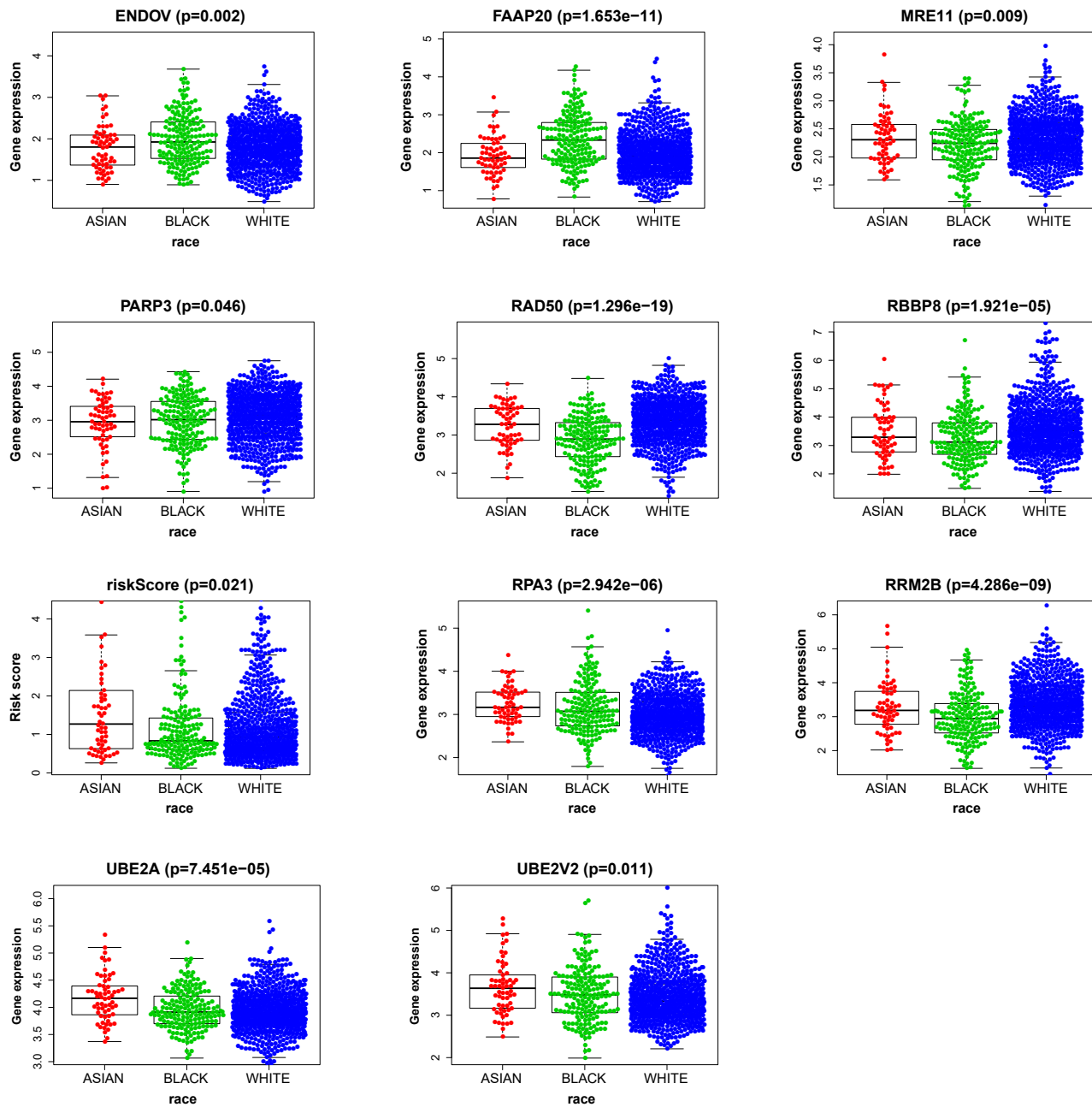**C**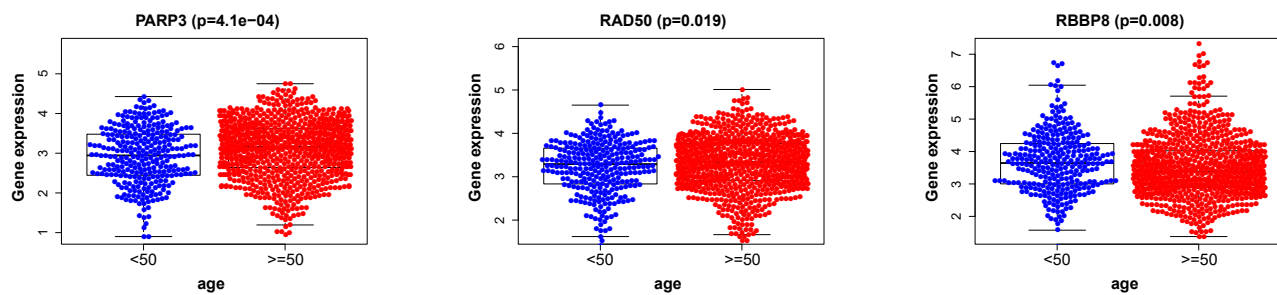

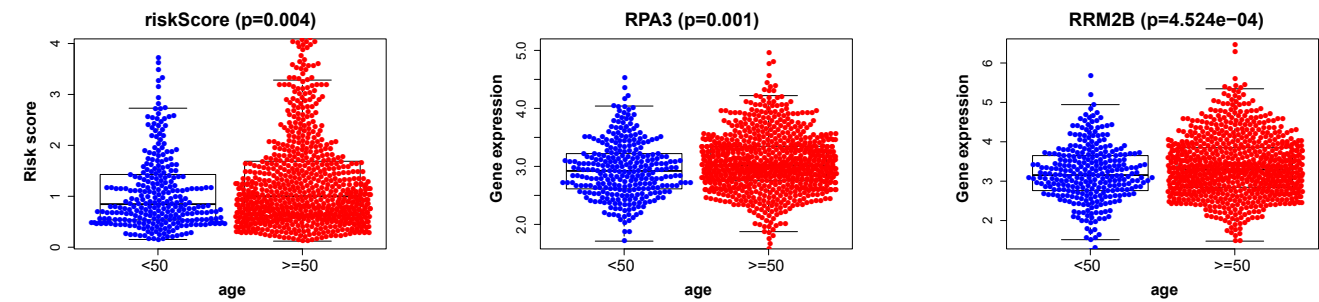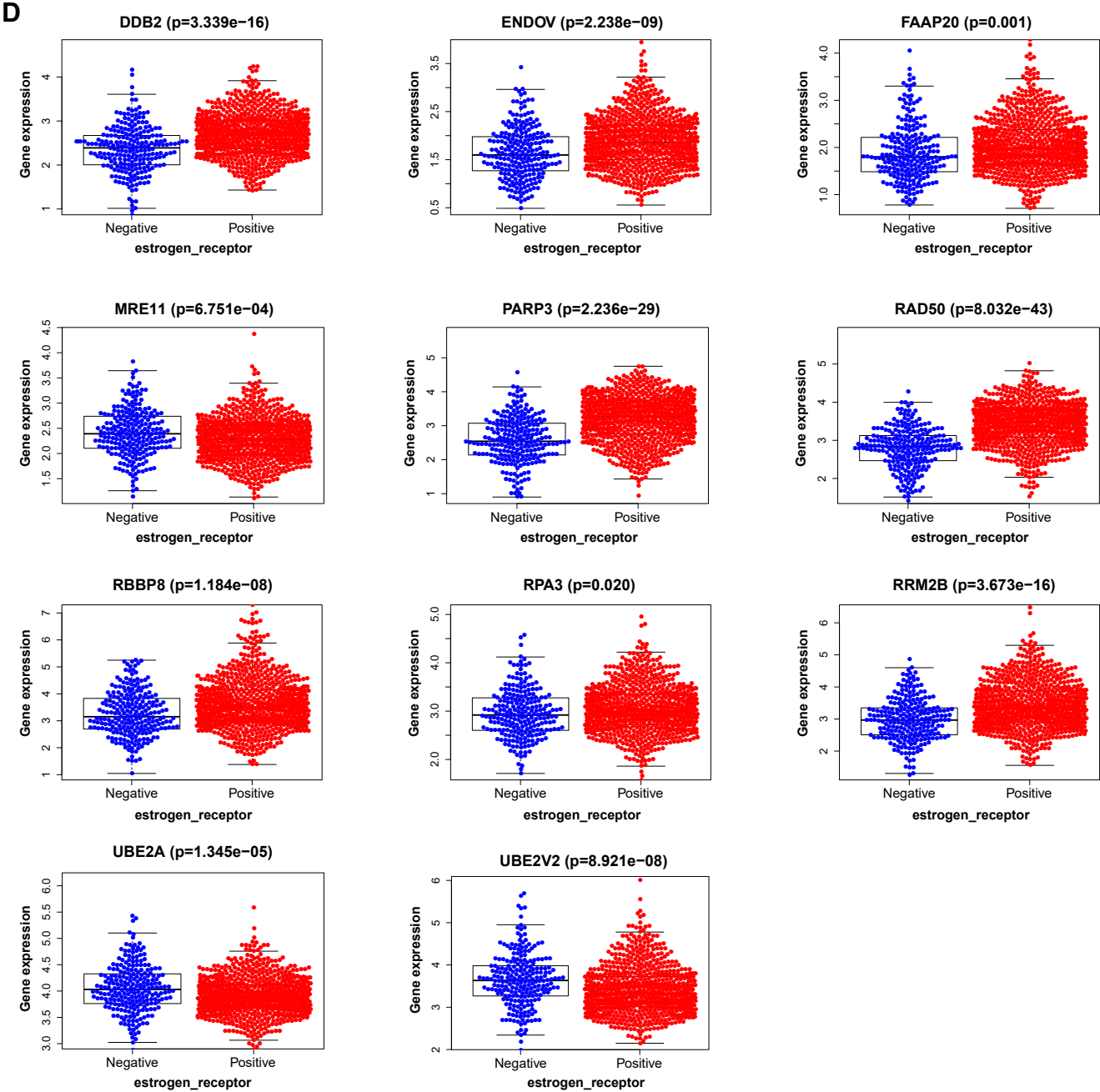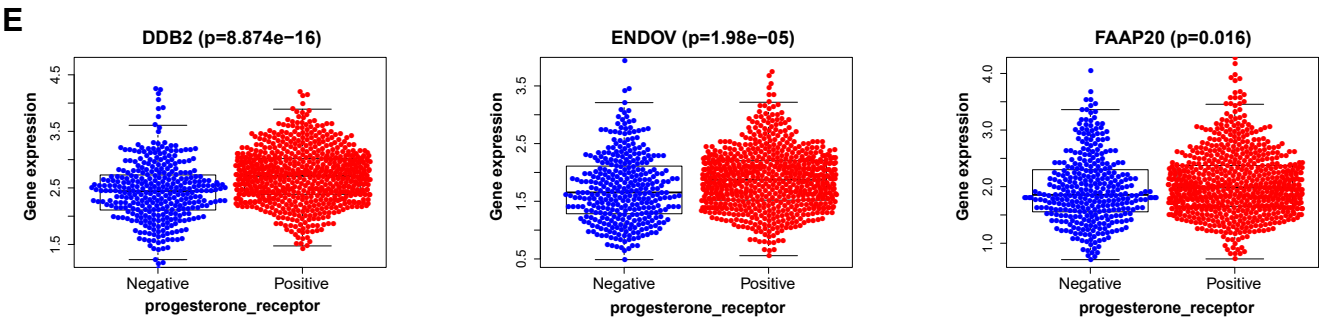

Continued

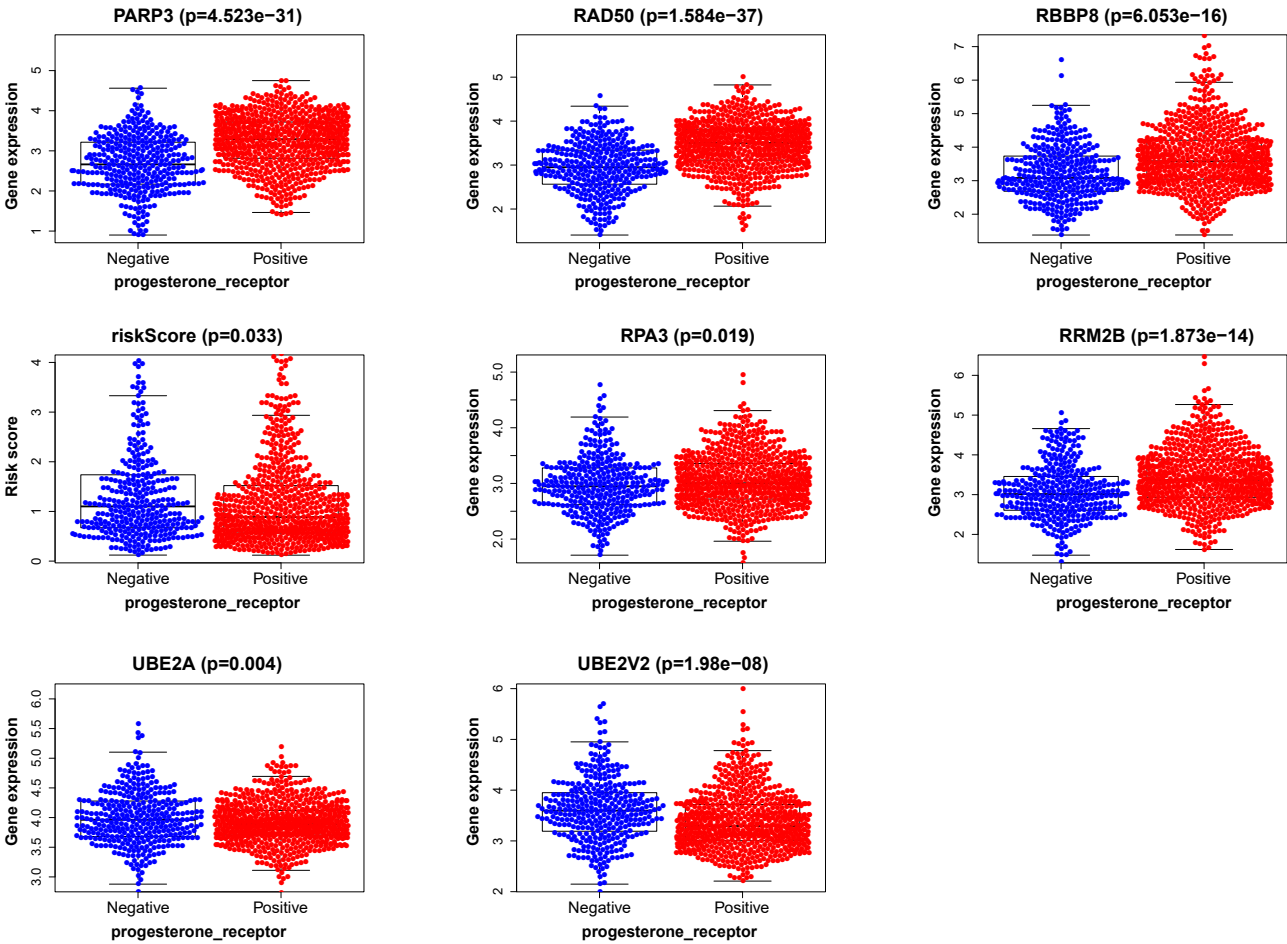

F

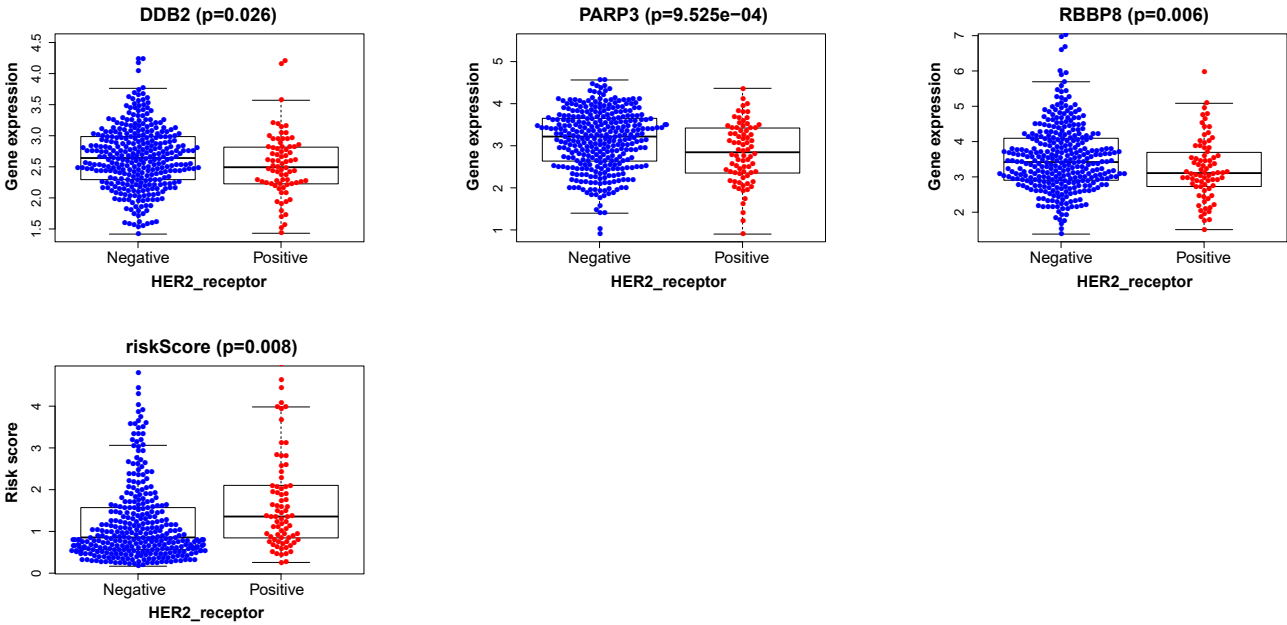

G

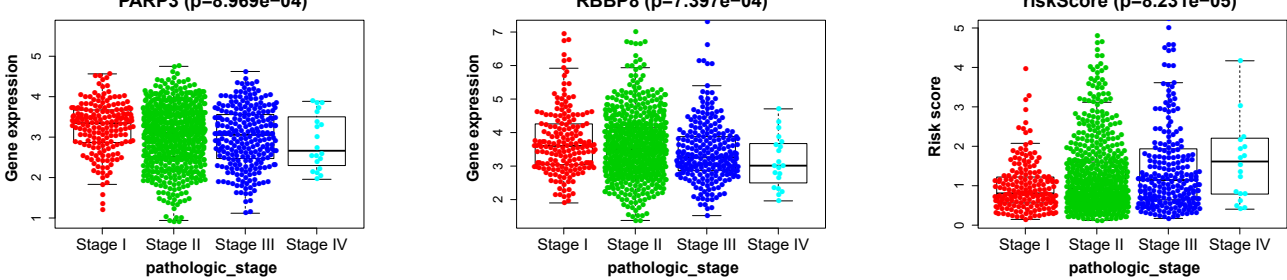

H

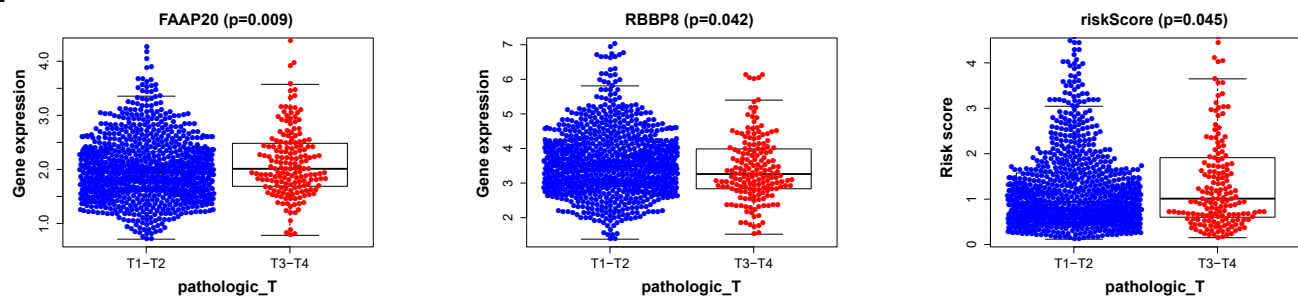

I

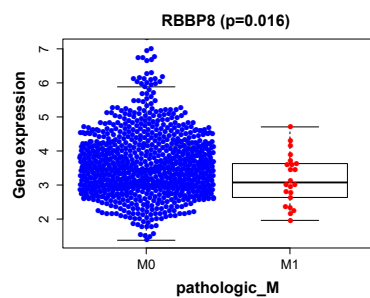

J

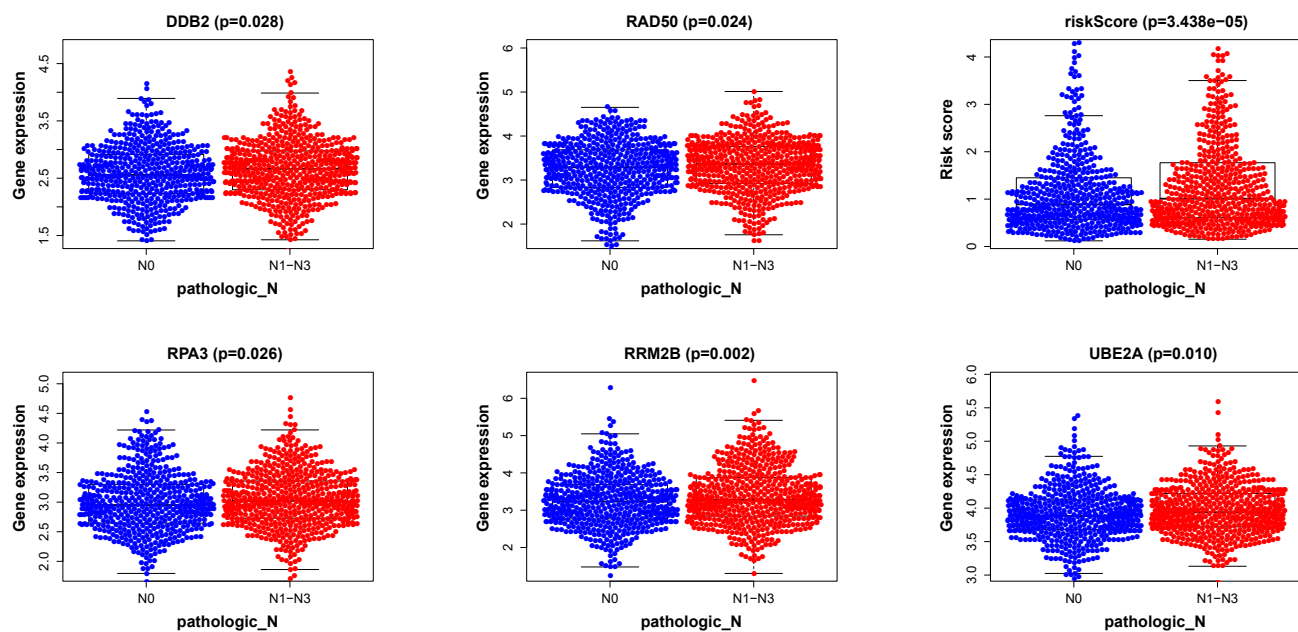

Supplement: Supplementary Figure 2 — Box plot for displaying relationship between prognostic DNA repair genes or risk score and clinical features. (A) Gender; (B) Race; (C) Age;(D) Estrogen receptor; (E) Progesterone receptor; (F) HER2 receptor; (G) Stage; (H) T stage; (I) M stage; (J) N stage. [file Image_2.pdf]

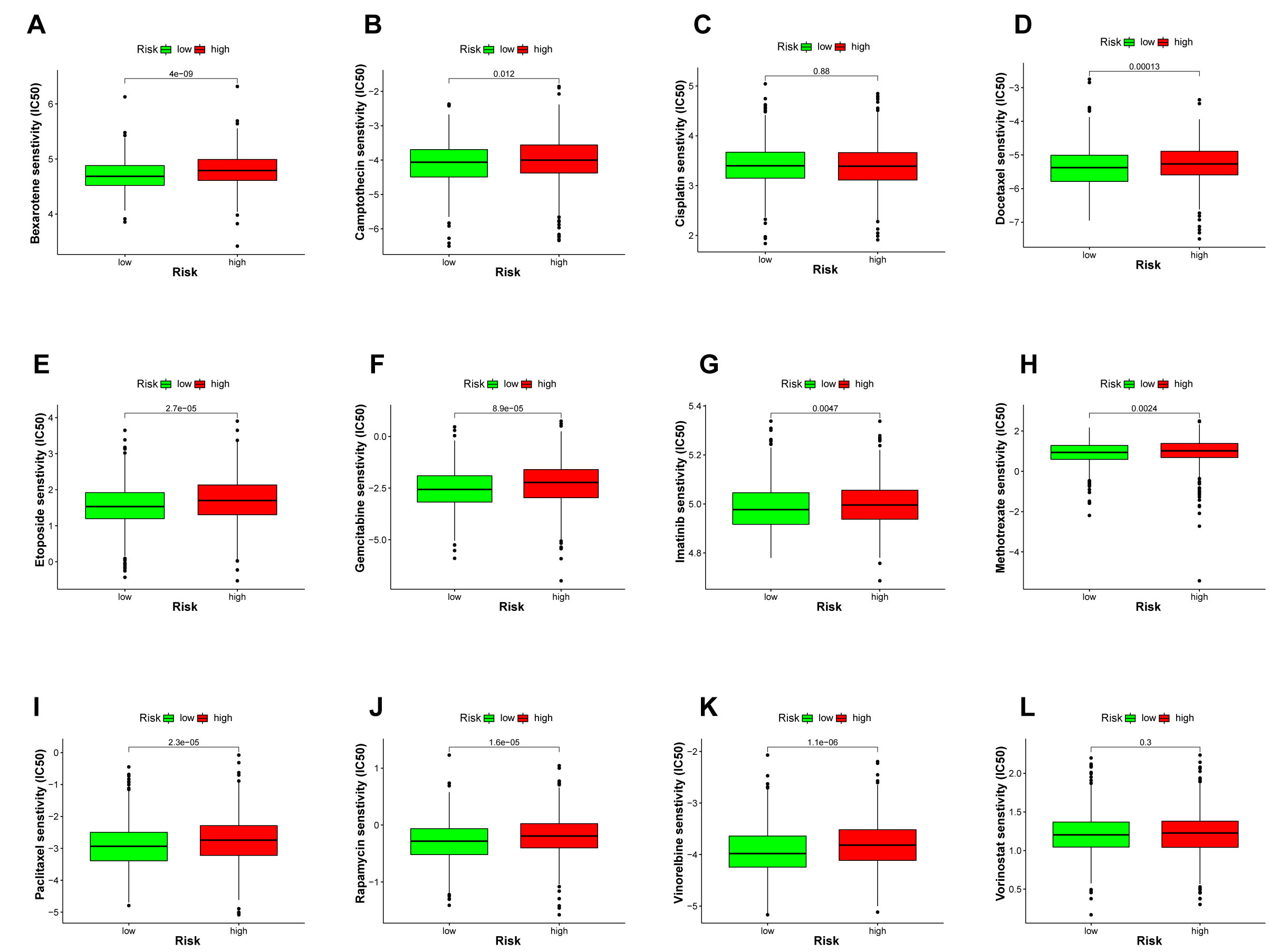

Supplement: Supplementary Figure 3 — The evaluated half maximal inhibitory concentration (IC50) for each of 16 anti-cancer drugs between high-risk group and low risk group were displayed in box plots. (A) Bexarotene; (B) Camptothecin; (C) Cisplatin; (D) Docetaxel; (E) Etoposide; (F) Gemcitabine;(G) Imatinib; (H) Methotrexate; (I) Paclitaxel; (J) Rapamycin; (K) Vinorelbine; (L) Vorinostat; Each dot stands for the estimated IC50 value of corresponding drug in breast cancer sample. The higher IC50 is the less sensitive breast cancer is to this drug. [file Image_3.jpeg]

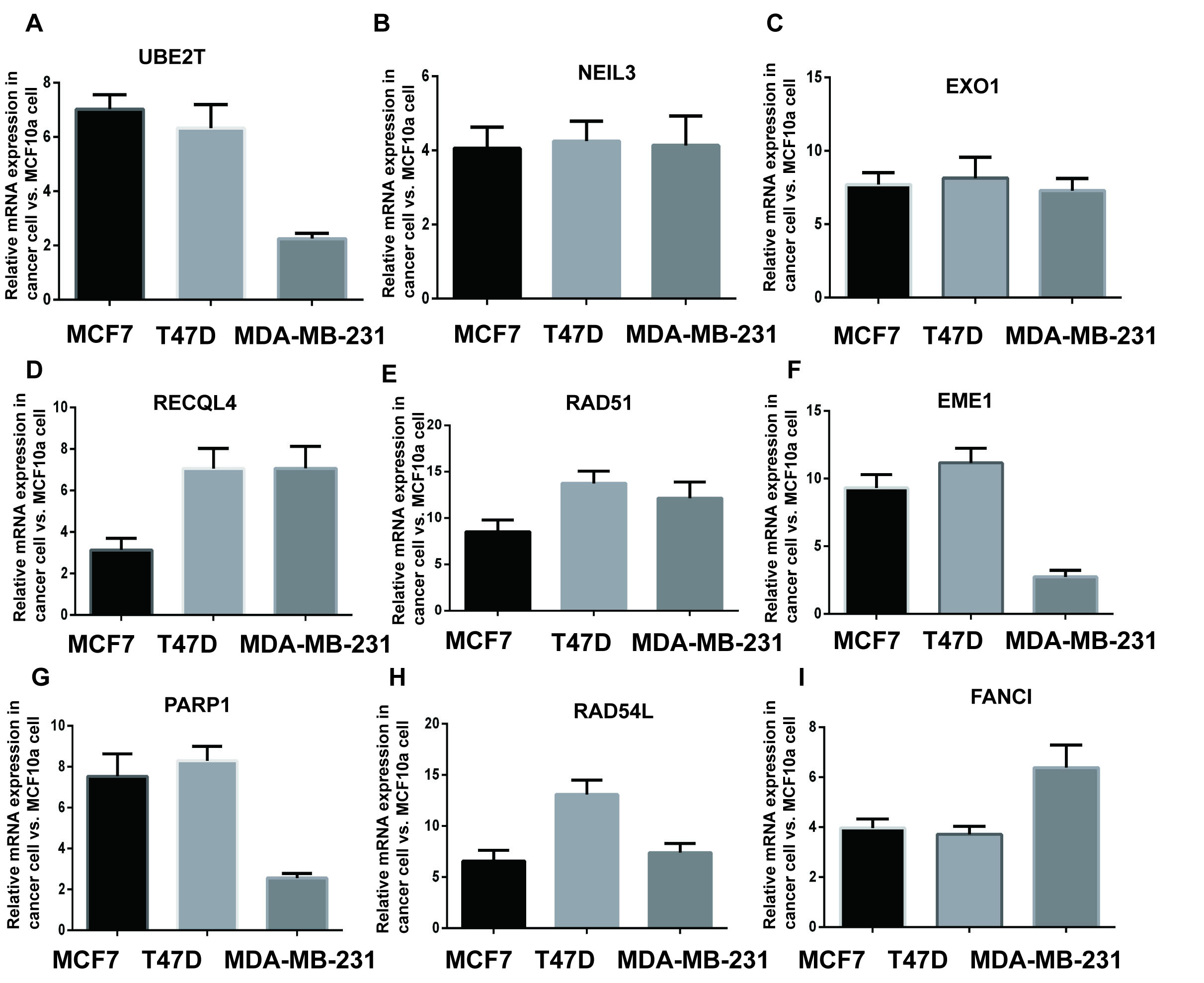

Supplement: Supplementary Figure 4 — RT-qPCR validation of relative expression level of differentially expressed DNA repair genes between breast cancer cell lines and normal breast cell line. Total RNA was isolated from breast cancer cell lines (MCF7, T47D and MDA-MB-231) and normal breast cell line (MCF-10a). Relative mRNA expression was analyzed by qPCR. (A) UBE2T; (B) NEIL3; (C) EXO1;(D) RECQL4; (E) RAD51; (F) EME1; (G) PARP1; (H) RAD54L; (I) FANCI. [file Image_4.jpeg]
